# The cnidarian *Hydractinia echinata* employs canonical and highly adapted histones to pack its DNA

**DOI:** 10.1186/s13072-016-0085-1

**Published:** 2016-09-06

**Authors:** Anna Török, Philipp H. Schiffer, Christine E. Schnitzler, Kris Ford, James C. Mullikin, Andreas D. Baxevanis, Antony Bacic, Uri Frank, Sebastian G. Gornik

**Affiliations:** 1 Centre for Chromosome Biology, School of Natural Sciences, National University of Ireland, Galway, Ireland; 2 Genetics Environment and Evolution, University College London, London, UK; 3 Division of Intramural Research, National Human Genome Research Institute, National Institutes of Health, Bethesda, MD 20892 USA; 4 Whitney Laboratory for Marine Bioscience, University of Florida, St. Augustine, FL 32080 USA; 5 Australian Research Council Centre of Excellence in Plant Cell Walls, School of Biosciences, The University of Melbourne, Parkville, VIC 3010 Australia; 6 NIH Intramural Sequencing Center, National Human Genome Research Institute, National Institutes of Health, Rockville, MD 20852 USA

**Keywords:** Histone, Chromatin, Cnidaria, Histone variants, Sperm-specific histones

## Abstract

**Background:**

Cnidarians are a group of early branching animals including corals, jellyfish and hydroids that are renowned for their high regenerative ability, growth plasticity and longevity. Because cnidarian genomes are conventional in terms of protein-coding genes, their remarkable features are likely a consequence of epigenetic regulation. To facilitate epigenetics research in cnidarians, we analysed the histone complement of the cnidarian model organism *Hydractinia echinata* using phylogenomics, proteomics, transcriptomics and mRNA in situ hybridisations.

**Results:**

We find that the *Hydractinia* genome encodes 19 histones and analyse their spatial expression patterns, genomic loci and replication-dependency. Alongside core and other replication-independent histone variants, we find several histone replication-dependent variants, including a rare replication-dependent H3.3, a female germ cell-specific H2A.X and an unusual set of five H2B variants, four of which are male germ cell-specific. We further confirm the absence of protamines in *Hydractinia*.

**Conclusions:**

Since no protamines are found in hydroids, we suggest that the novel H2B variants are pivotal for sperm DNA packaging in this class of Cnidaria. This study adds to the limited number of full histone gene complements available in animals and sets a comprehensive framework for future studies on the role of histones and their post-translational modifications in cnidarian epigenetics. Finally, it provides insight into the evolution of spermatogenesis.

**Electronic supplementary material:**

The online version of this article (doi:10.1186/s13072-016-0085-1) contains supplementary material, which is available to authorized users.

## Background

Most eukaryotes package and order their nuclear DNA into chromatin using a class of proteins called histones [1–4]. Histones evolved in the common ancestor of Archaea and Eukaryota, as evidenced by structural homology between modern eukaryotic and archaeal histones [5, 6]. To facilitate packaging, the histones form an octameric core complex containing two of each of the four core histones (H2A, H2B, H3 and H4); in turn, DNA wraps around this histone core complex to form the basic unit of chromatin compaction called nucleosome [4, 7]. Nucleosomes compact the genome while still providing dynamic access for processes such as DNA transcription, replication and repair. To bring about these regulatory functions, a diverse array of distinct, combinatorial post-translational modifications occurs on tail domains of histones [8, 9]. To date, hundreds of epigenetically active histone modifications have been identified, for example monoubiquitination, acetylation, mono-, di- and tri-methylation of lysines, as well as mono- and dimethylation of arginines, phosphorylation of serines, threonines and tyrosines, and isomerisation of prolines [10].

In many eukaryotes canonical histone expression is replication-dependent, occurring in S-phase. Metazoans have evolved a unique mechanism to achieve a timely and highly coordinated expression of histones during replication. For this, they use non-polyadenylated mRNAs with a specific 3′-untranslated region (UTR) containing a stem-loop sequence of 26 bp followed by a purine-rich downstream element. This region of the mRNA sequence facilitates histone mRNA maturation by binding U7 small nuclear RNA and a specific cleavage complex. Stem-loop recognition and mRNA stabilisation are cyclin-dependent and tightly linked to S-phase [11]. The genes of core histones do not contain introns, and their mRNAs generally have short 5′- and 3′-UTRs.

Due to their importance for essential functions such as DNA packaging and controlling DNA access, histones are amongst the most conserved and slowest evolving proteins known in eukaryotes [12]. Alongside the core histones, several types of histone variants have evolved to fulfil specific roles in diverse but essential functions such as chromosome segregation, meiotic recombination, transcriptional regulation and DNA repair [1, 6]. The emergence of many of these variants, such as CENP-A (chromosome segregation), H3.3 (transcription control), H2A.Z (promoter activation) and H2A.X (DNA repair), dates back to the earliest known diversifications of all extant eukaryotic lineages. As a consequence, these variants are of near universal occurrence amongst all eukaryotes [6, 13]. Other histones, such as the sperm-specific H2B histones found in sea urchins, and an oocyte-specific H2A.X variant in frogs, evolved later and have a more limited distribution. They carry out distinctive functions reflecting the unique biology of their host organisms [14, 15].

Protamines are histone-related, arginine-rich sperm nuclear basic proteins (SNBPs) that replace histones in the nuclei of the sperm of many animals to achieve a high level of DNA condensation. They are grouped into three types: histone type (H-type), protamine-like (PL-type) and protamine type (P-type). All three types may co-occur in different animal clades. The evolution of protamines is not well understood, but they are thought to be derived from histone H1 [16–19].

The Cnidaria are the sister group to Bilateria [20–22]. Besides their key phylogenetic position, cnidarians embody unique features that include remarkable regenerative powers and longevity, making them interesting research subjects for studying regeneration and ageing [23, 24]. Cnidarian stem cells were the first to be studied in any animal [25]. Many cnidarians can regenerate all tissue types and is generally immune to tumorigenesis [23, 26, 27]. It is likely that many of the unique biological features of cnidarians will depend on chromatin packaging properties and epigenetic regulation; however, the literature on cnidarian epigenetics and histones is fragmentary and incomplete.

Here, we present the full histone gene complement of the hydrozoan, colony-forming cnidarian *Hydractinia echinata* and discover that protamines are absent in this species. We analyse the genomic loci of all *Hydractinia* histones and show their spatial and temporal expression patterns at mRNA and protein levels. We place particular emphasis on histone variants found in the *Hydractinia* genome and discuss their potential evolutionary and functional contexts.

## Methods

### Animal culture

*Hydractinia echinata* colonies were collected from Galway Bay (Ireland) or Roscoff (France). The animals were cultured in artificial seawater at 18 °C under 14-/10-h light–dark regimes and were fed *Artemia franciscana* nauplii four times a week and ground oyster once per week. The animals spawn daily [28]. Polyps were harvested from mature colonies.

### Genomic DNA extractions

Genomic DNA was extracted from adult female feeding polyps. Polyps were separated from colonies using surgical scissors and repeatedly washed in sterile-filtered artificial seawater. The animal tissue was then disrupted in 1 ml of DNA lysis buffer (100 mM Tris HCl (pH8), 1 % SDS, 50 mM EDTA) using a plastic pestle. Thereafter, 2 µl each of RNaseA and RNaseT1 (both Thermo Fisher) were added and incubated for 1 h at 37 °C. Following this, 2 µl of proteinase K (25 mg ml^−1^, Qiagen) were added and the solution was further incubated at 50 °C for 2 h. Finally, DNA was isolated using equal amounts of phenol (pH 8) and chloroform, and chloroform clean-up. Genomic DNA was precipitated from the aqueous phase using 1/10 volume of 5 M NaCl and 2.5 volume of ethanol and washed in 70 % ethanol three times. The resulting pellet was air-dried at room temperature and resuspended in Tris/EDTA (10 mM/1 mM, pH 8.0).

### Genome sequencing and preliminary assembly

From genomic DNA a draft assembly was generated as follows: a paired-end Illumina fragment library was generated following established protocols (Illumina, Inc) and sequenced on a single MiSeq lane; 8,821,453 million read pairs were then assembled into 126,814 contigs (contig N50 = 4.9 kb) using the Phusion assembler [29]. Subsequently, two mate-pair DNA libraries with insert sizes of 3.4 and 5.5 kb from the same genomic source were constructed and sequenced on two lanes of HiSeq Rapid Run Illumina sequencing, producing 75,388,716 and 98,052,384 reads, respectively. These reads were used to order and orient the contigs into 77,987 scaffolds (scaffold N50 = 63.8 kb) using the Phusion assembler. The final assembly was 421 Mb. The raw reads are deposited into the NCBI Short Read Archive (accession numbers SRX1879642, SRX1879940 and SRX1880157).

### RNA extraction, sequencing, RNA mapping and transcriptome assembly

For life stage-specific RNA read mapping and transcriptome assemblies, RNA was extracted from adult male and female sexual polyps, adult feeding polyps and 48-h old larva. Any contaminating material not representing the selected stage was removed from the samples before processing, while seawater was replaced by three washes in sterile 0.5 M NaCl. Total RNA was isolated by guanidinium thiocyanate and CsCl cushion ultracentrifugation [30]. Standard cDNA synthesis was performed by the Cologne Center for Genomics at the University of Cologne. A total of 100-bp paired-end reads (170 bp insert size) were sequenced on Illumina HiSeq machines. The software FastQC (http://www.bioinformatics.bbsrc.ac.uk/projects/fastqc/ {last accessed 07/06/2016]) was used to assess data quality and trimmomatic [31] to clean the reads. The clc mapper (CLC Bio software, Qiagen) was used to map RNA-Seq data against genomic contigs containing the histone cluster and analyse coverage for the different genes. BAM files containing the mapping data can be accessed online at https://dx.doi.org/10.6084/m9.figshare.3436460.v1. A *Hydractinia* transcriptome using RNA extracted from adult female feeding polyps (see above) was generated using Trinity (v2.0.6; [32]) from raw reads and clustered using CD-HIT-EST and CAP3 as described in [33]. Following assembly and clustering, ORFs were predicted using EMBOSS *getorf* (>200 amino acids (-minsize 300), from START to STOP codons (-find 1); http://emboss.sourceforge.net/ [last accessed: 20/04/2016]). The longest ORF per transcript was retained.

### Histone searches, histone gene loci annotation and visualisation of bioinformatics data

Transcripts and genomic loci sequences, which contained histone genes, were identified using NCBI BLAST from the transcriptome or genome draft assembly, respectively, and extracted using Geneious R8 (Biomatters). Genomic sequences were then annotated using the MAKER2 pipeline [34] at standard settings. RNA-Seq data, transcriptome and protein evidence (EMBOSS longest ORFs) were supplied to MAKER2 to produce evidence-based genome annotations in gff3 format. MAKER2 was set to use the ab initio predictors SNAP, GeneMark and Augustus to optimise annotations (for references of software used see [34]). A *Brugia malayi* protein model was used in Augustus since this model has empirically shown to be superior to newly generated models trained on *Hydractinia* protein data sets. Both *Hydractinia echinata* and *Brugia malayi* genomes are AT-rich, and it is assumed that the more exhaustive protein data available for *Brugia* resulted in the superior performance of the *Brugia* model in predicting protein-coding regions in such an AT-rich environment. Following MAKER2 annotation, genomic loci of histone genes were defined as genomic regions that contain the gene of interest and extend to the STOP/START of the coding sequence of the neighbouring up- and downstream genes. Histone genomic loci sequences, exons from MAKER2 gene models and stage-specific RNA-Seq alignments in bam format were transformed into data tracks and visualised using the R package Gviz (version 1.15.6; [35]). Histone 3′-UTR stem-loop and histone cluster Arginine tRNA structures were predicted using ‘RNAfold’ within the ViennaRNA Package software (http://www.tbi.univie.ac.at/RNA/ [last accessed: 20/04/2016]) and completed in Illustrator CS6 (Adobe). A k-mer-based alignment-free sequence comparison was performed using kmacs (http://kmacs.gobics.de/ [last accessed: 20/04/2016]).

### Copy estimation of the canonical histone repeat cluster

A phrap assembly (http://www.phrap.org/phredphrapconsed.html; phrap version 1.090518) was generated from a randomly selected subset of Illumina HiSeq reads (40,000 paired-end 250 base reads), from an Illumina TruSeq DNA PCR-free library of *H. echinata* gDNA using the following command: phrap -ace test20kreadpairs -retain_duplicates -minscore 140 -minmatch 70 -vector_bound 0 -repeat_stringency .999 -forcelevel 0. The largest contig, out of a total of 11,790 contigs, included a complete representation of one example copy of the histone region (5998 bases). The second and third largest contigs were joined together in consed [36] using overlap information forming a complete representation of one example copy of the ribosomal DNA (rDNA) repeat region (7039 bases). Using a 17-base-long k-mer word use histogram from 31.1 × 10^6^ paired-end 250 base reads, k-mers from the histone region appear at approximately 28,000-fold coverage and the rDNA repeat region appears at the approximately 46,000-fold coverage (Additional file 1: S1B). With the diploid peak at 20× coverage (Additional file 1: S1A), this indicates there are 1400 copies of the histone region and 2300 copies of the rDNA repeat region in a diploid nucleus.

### Gene phylogenies

*Hydractinia* histone sequences were obtained as described above. *Aiptasia* (*Exaiptasia pallida*) histones sequences were extracted from a database downloaded from http://aiptasia.reefgenomics.org/ [last accessed: 14/06/2016; see also GenBank accession PRJNA261862]. Other histone sequences were extracted from GenBank, and their accessions are given in Additional file 3: S3. Protein alignments were generated using MAFFT (v1.3.3; [37]), and ambiguous sites were manually removed using Geneious (version R8; Biomatters). Maximum likelihood phylogenies were performed using a public, Web-based RAxML server [38] using a standard, empirical JTT (Jones, Taylor and Thornton) substitution matrix. The best-scoring tree was visualised using FigTree (v1.4.2; http://tree.bio.ed.ac.uk/software/figtree) and annotated in Illustrator CS6 (Adobe).

### In situ hybridisation, EdU-labelling, FISH and microscopy

For in situ hybridisation (ISH) experiments, male and female sexual and feeding polyps were cut from adult colonies using surgical scissors, anaesthetised for 30 min in 4 % MgCl in seawater and fixed in 4 % paraformaldehyde. *In situ* hybridisation was performed as previously described [39, 40]. Hybridisations were performed at 50 °C. DNA templates for RNA probe synthesis were obtained by PCR from cDNA or genomic DNA (for single-exon histone genes) using gene-specific primers (Additional file 2: S2). T7 and SP6 RNA promoters were added to the 5′ ends of the primers when generating probes. ISH and fluorescent ISH (FISH) probes were digoxigenin (Dig) or fluorescein (FITC) labelled using SP6 or T7 RNA polymerase kits (both Fermentas), respectively, according to the manufacturer’s instructions. Antibodies and dilutions for ISH and FISH were the following: anti-Dig AP (Roche 11093274910, 1:1000); anti-FITC AP (Roche 11426338910, 1:1000); anti-Dig conjugated to horseradish peroxidase (POD; Roche 11207733910, 1:1000) and anti-FITC POD (Roche 11426346910, 1:1000). The Tyramide Signal Amplification kit (PerkinElmer) was used for FISH according to the manufacture’s instructions. EdU incorporation was performed for 30 min at a concentration of 150 μM. Following this, FISH was performed as described above. For EdU visualisation, animals were processed using the Click-iT EdU AlexaFluor 488 Imaging kit (Life Technologies) according to the manufacturer’s instructions. ISH images were acquired on an Olympus BX51 inverted microscope, and FISH images were taken on an Olympus FV1000 inverted confocal microscope.

### MNase assay

MNase assays were carried on *Hydractinia echinata* sperm. To do so, male polyps were first cut from adult colonies using surgical scissors. Then sperm were extracted from approximately 60 mature gonads using a fine syringe needle (23^5^/_8_″G) into 20 µl of 4 % MgCl_2_ · 6H_2_O (w/v). Upon extraction 1 ml of hypotonic lysis buffer (10 mM DTT, complete protease inhibitor (Roche)) was added and the samples were incubated for 30 min on ice. Nuclei were centrifuged for 25 min at 16,000×*g* at 4 °C. The nuclei-containing pellet was then resuspended in 800 µl of chromatin digestion buffer (20 mM Tris [pH 7.5], 15 mM NaCl, 60 mM KCl, 1 mM CaCl_2_, 5 mM MgCl_2_, 300 mM sucrose and 0.4 % NP40 containing 0.0125 units of RNAse T1 (Thermo Fisher)). The suspension was separated into four 200 µl aliquots. Aliquots were warmed to 37 °C for 1 min in a PCR machine with lid temperature of 42 °C. Then 0, 0.02, 0.2 and 0.4 units of MNase (NEB) were added, mixed and incubated at 37 °C for a further 3 min. The reaction was stopped with 0.2× volumes (6.8 µl) 100 mM EDTA and 4 % SDS. Five microlitres of proteinase K (20 µg µl^−1^, Qiagen) was then added. The solution was then incubated at 55 °C for 1 h, phenol–chloroform-extracted, dissolved in 15 µl nuclease-free H_2_O, run on a 2 % agarose gel, containing SYBRSafe DNA-stain, at 100 V for 40 min and visualised using a MultiImage^2^ (Alpha Innotech) UV box.

### Acid extraction of sperm proteins and SDS-PAGE

Acid-soluble proteins were extracted from nuclei-enriched fractions of *Hydractinia echinata* sperm. To do so, male polyps were first cut from adult colonies using surgical scissors. Then the mature gonads were cut from these polyps, transferred into a 1.5-ml Eppendorf tube containing 500 µl of sterile-filtered artificial seawater (Instant Ocean) and squeezed using a sterile pestle resulting in sperm release. Sperm were then pelleted at 100×*g* at 4 °C for 2 min and washed twice in sterile seawater. The sperm pellet was then resuspended in nuclear extraction buffer (10 mM Tris–Cl [pH 8.0], 1 mM KCl, 1.5 mM MgCl_2_ and 1 mM DTT in complete protease inhibitor cocktail [Roche]) and incubated for 30 min on ice. This results in sperm rupture and release of sperm nuclei. Nuclei were spun out of the suspension at 16,000×*g* at 4 °C for 10 min. The supernatant was discarded, nuclei resuspended in 400 µl of 0.4 N H_2_SO_4_ and incubated with slow rotation overnight at 4 °C. Insoluble material was pelleted at 16,000×*g* for 10 min at 4 °C, and soluble proteins were precipitated for 2 h on ice using 132 µl of trichloroacetic acid (TCA; 100 %, w/v), washed twice in ice-cold acetone, air-dried and dissolved in 100 µl of protease-free water. SDS-PAGE was performed with 10 µg protein using 4–12 % Bis–Tris gradient pre-cast gels (Novex) according to the manufacturer’s instructions. Gels were stained using Coomassie blue and recorded using a MultiImage^2^ (Alpha Innotech) gel chamber.

### Protein mass spectrometry

Protein bands were excised using fresh sterile scalpel blades (one blade per band), transferred to 1.5-ml Eppendorf tubes, frozen at −80 °C and finally lyophilised at −70 °C under vacuum for shipment. Whole acid extracts of sperm were similarly lyophilised. Upon arrival at the proteomics facility (The Plant Cell Biology Research Centre, School of BioSciences, The University of Melbourne, Australia), lyophilised SDS-PAGE bands and whole acid extracts were rehydrated in 100 mM ammonium bicarbonate for 5 min and digested as described in [41]. Following digestion, samples were resuspended in 20 µl of 0.1 % formic acid and 3 µl of each sample was analysed on a Q Exactive Plus (Thermo Fisher) coupled to an Ultimate 3000 RSLC nanosytem (Dionex). The nanoLC system was equipped with an Acclaim Pepmap nano-trap column (Dionex) and an Acclaim Pepmap analytical column (Dionex), operating at a flow rate of 3 µl min^−1^ with a 40-min gradient of 3–80 % acetonitrile containing 0.1 % formic acid. The Q Exactive Plus mass spectrometer was operated in positive mode, spray voltage was set to 1800 kV, S-lens RF level at 50 and heated capillary at 250 °C. Peptides were fragmented using normalised collision energy of 35 and activation time of 0.1 ms in the data-dependent mode, whereby the top 10 ions between 400 and 1600 m/z with a charge state between 2+ and 5+ were selected for MS/MS. The MS data were analysed using MASCOT version 2.4 (Matrix Science) search engine against the transcriptome with the following parameters; enzyme: trypsin; fixed modifications: carbamidomethyl (C); variable modifications: acetylation (K), MS peptide tolerance: 10 ppm, MS/MS tolerance: 0.1 Da, number of missed cleavages: up to 1. Only proteins with two or more peptides with a *p* < 0.05 were considered present, after satisfying manual inspection.

## Results

### The *Hydractinia* genome encodes 19 histones and no protamines

**Fig. 1:**
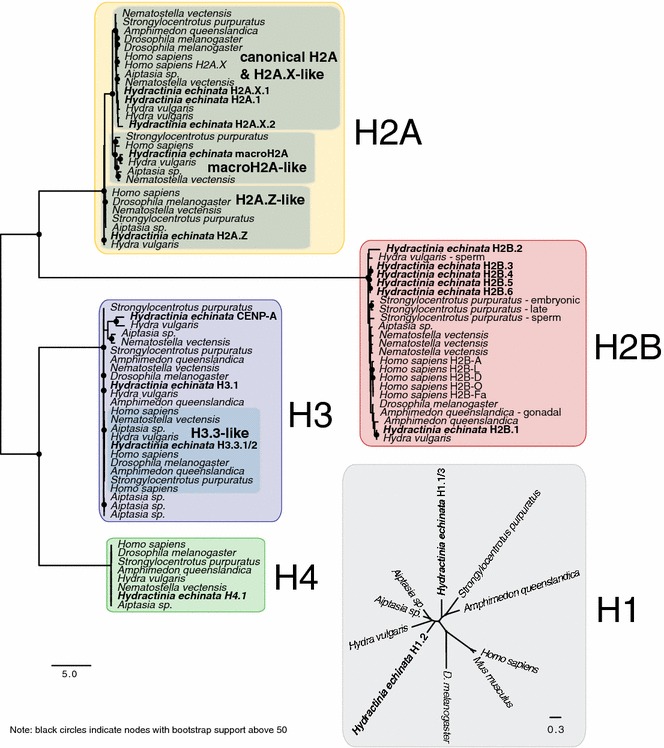
Unrooted maximum likelihood phylogenies of *Hydractinia echinata* histones. *Black circles* indicate nodes with bootstrap support above 50. H2A.Z and macroH2A and H3.3 variants from clear clades, whereas H2A.X, all H2B variants and CENP-A (H3-derived) are not clearly separated from their canonical counterparts. The H1 phylogeny is unresolved

**Fig. 2:**
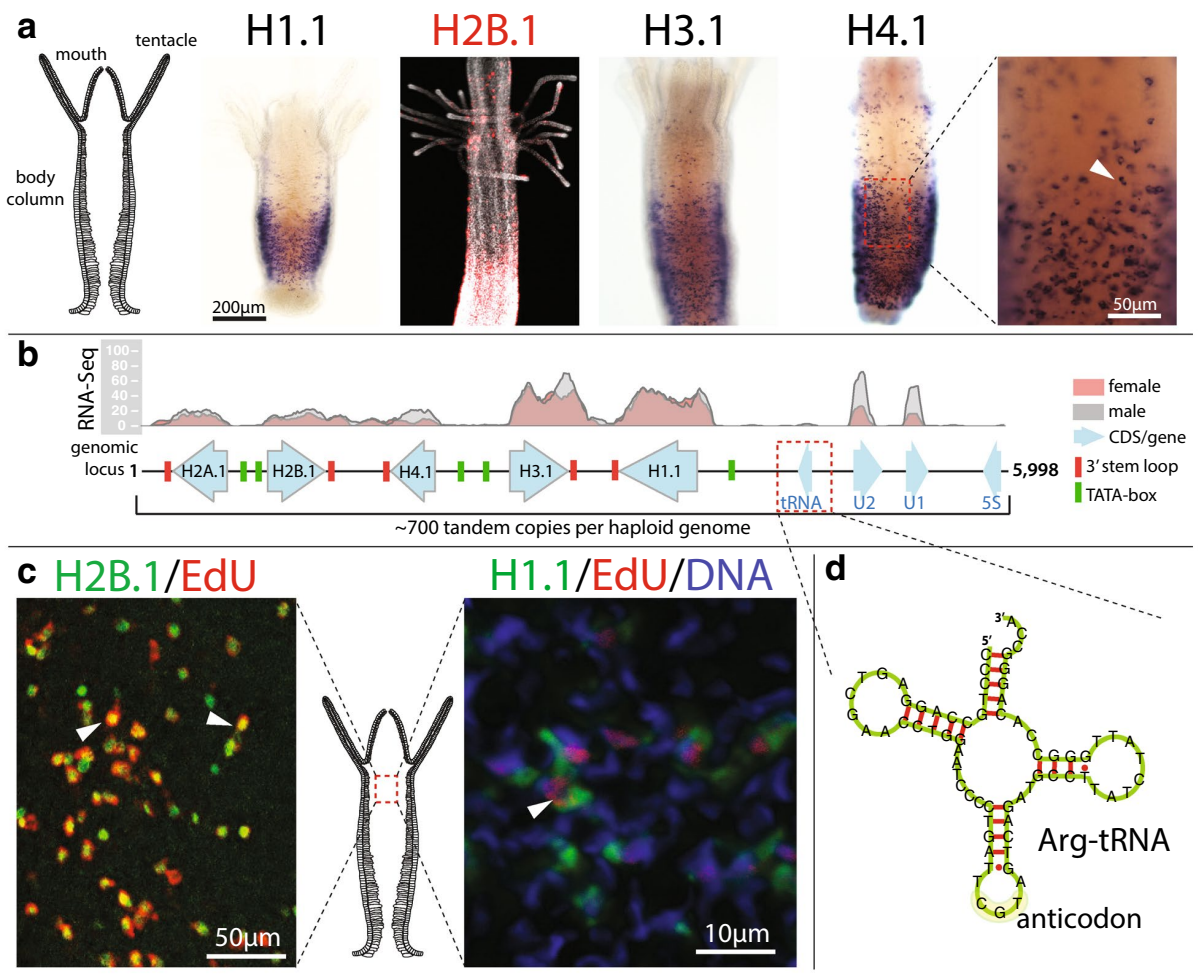
Annotated genomic locus and expression profiles of the H1.1 and core histones H2A.1, H2B.1, H3.1 and H4.1 of *Hydractinia echinata*. **a** Expression of H1.1, H2B.1, H3.1 and H4.1 in feeding polyps. H2B.1 was analysed using a red fluorescent probe. The* white wedge* highlights an individual cell expressing H4.1. **b** Organisation of the genomic locus depicting coding sequences, mapped RNA reads (showing the number of reads mapped), predicted TATA-boxes and 3′-UTR stem-loops. Extended RNA read mapping can be found in Additional file 6: S6. **c** Co-localisation of gene expression (*green*) and S-phase (EdU, *red*). A *white wedge* highlights histone-expressing cells in S-phase. **d** Predicted structure of the histone cluster arginine tRNA

We identified a total of 19 histones in the genome of *Hydractinia echinata* using sequence alignment and phylogenomics. These include three H1 genes, one H2A, two H2A.X, one H2A.Z, one macroH2A, six H2B, three H3 genes, one CENP-A and one H4 (Fig. 1; Table 1; Additional file 3: S3, Additional file 4: S4). All 19 genes possess a TATA-box (consensus TAWAAA) upstream of the coding sequence. Eighteen of the 19 genes are expressed in one or more stages throughout the life cycle as shown by RNA-Seq, in situ hybridisation (ISH) or MS-based proteomic techniques (Figs. 2, 4, 5, 6, 7; Additional file 4: S4, Additional file 5: S5). The remaining H1.3 gene is probably a pseudogene. Neither protamine (P-type) nor protamine-like (PL-type) genes were found, consistent with previous studies in other hydrozoans [16, 42, 43] (Fig. 7f, Additional file 9: S9).

**Table 1:** *Hydractinia echinata* histone complement

	Expression pattern	Replication-dependency?	Histone type	Genomic locus
H1.1	Male, female, feeding polyps	Y^§^	Core histone	1 exon, canonical cluster incl. H1, H2A, H2B, H3, H4
H1.2	Male, female, feeding polyps	N^§^	*Hydractinia* variant	Single locus, 2 exon
H1.3	–	–	*Pseudogene*	Single locus, 1 exon
H2A.1	*Transcripts present in all life stages analysed*	Y^§§^	Core histone	1 exon, canonical cluster incl. H1, H2A, H2B, H3, H4
H2A.Z	Male, female, feeding polyps	N	Replication-independent variant	Single locus, 5 exons
H2A.X.1	Male, female, feeding polyps	N	Replication-independent variant	Single locus, 2 exons
H2A.X.2	Female germ cells	N	*Hydractinia* variant	Single locus, 5 exons
macroH2A	*Transcripts present in all life stages analysed*	N	Replication-independent variant	Single locus, 8 exons
H2B.1	Male, female, feeding polyps	Y^§^	Core histone	1 exon, canonical cluster incl. H1, H2A, H2B, H3, H4
H2B.2	*Transcripts present in male polyps only*	Y^§§^	*Hydractinia* variant	1 exon, germ cell-specific cluster incl. H2B.2, H2B.5 H2B.6
H2B.3	Male germ cells	Y^§^	*Hydractinia* variant	Single locus, 1 exon
H2B.4	Embryo, male, female, feeding polyps	Y^§^	*Hydractinia* variant	Single locus, 1 exon
H2B.5	Male germ cells	Y^§§^	*Hydractinia* variant	1 exon, germ cell-specific cluster incl. H2B.2, H2B.5 H2B.6
H2B.6	Male germ cells	Y^§§^	*Hydractinia* variant	1 exon, germ cell-specific cluster incl. H2B.2, H2B.5 H2B.6
H3.3.1	Male, female, feeding polyps	N^§^	Replication-independent variant	Single locus, 2 exons
H3.3.2	Male, female, feeding polyps	Y^§^	Replication-dependent *Hydractinia* and Echinoderm variant	Single locus, 1 exon
H3.1	Male, female, feeding polyps	Y^§§^	Core histone	1 exon, canonical cluster incl. H1, H2A, H2B, H3, H4
CENP-A	*Transcripts present in all life stages, highest in male polyps*	N	Replication-independent variant	Single locus, 2 exons
H4.1	Male, female, feeding polyps	Y^§§^	Core histone	Canonical cluster incl. H1, H2A, H2B, H3, H4

^§^ Confirmed using EdU incorporation

^§§^ 3′-UTR stem-loop present

### Canonical histones

**Fig. 3:**
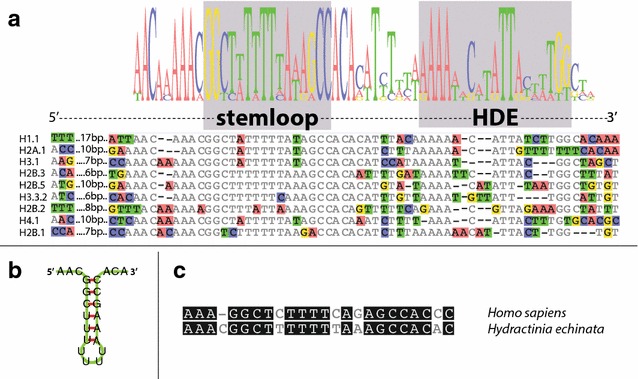
Analysis of the 3′-UTR stem-loop of *Hydractinia echinata* histone mRNAs. **a** Stem-loop sequence alignment, consensus sequence and sequence logo. Nucleotide sequences start after the termination codon (not shown), and 6–17 non-conserved base pairs are omitted before the stem-loop sequences begin. The alignment continues past the histone downstream element (HDE). Sequence differences are highlighted. **b** Predicted structure of the *Hydractinia echinata* histone 3′-UTR stem-loop, **c** Comparison of the human and *Hydractinia echinata* histone 3′-UTR stem-loop consensus sequences

RNA-Seq showed that H1.1, H2A.1, H2B.1, H3.1 and H4.1 are expressed in all life stages analysed (Additional file 6: S6). The corresponding proteins can be readily identified by MS-based proteomics analysis in acid extracts from adult feeding polyps, sexual polyps and larva (Table 1; Additional file 4: S4, Additional file 5: S5). These histones are organised as single-exon genes in a 5998-bp-long tandem repeat cluster (Fig. 2b). Interestingly, this cluster also contains a 5S rRNA, a U1 and U2 snRNA and an Arg-tRNA (GCA codon) gene (Fig. 2d); a constellation not previously described. Both snRNAs and the 5SrRNA genes are transcribed across all life stages investigated (Fig. 2b, Additional file 6: S6). The exact number of canonical histone cluster repeats is undetermined due to the repetitive nature of the locus, but estimations based on a k-mer depth histogram suggest at least 700 clusters per haplotype (Additional file 1: S1). ISH shows that H1.1, H2A.1, H2B.1, H3.1 and H4.1 are expressed in a band-like pattern in polyps in an area that is known to harbour the majority of proliferative cells (Bradshaw et al. [24]). The 3′-UTRs of these histones lack a polyadenylation (polyA) signal and instead contain a highly conserved 16-bp stem-loop structure as well as a purine-rich histone downstream element (HDE; Fig. 3a–c). Both the stem-loop and the HDE are hallmark signs of replication-dependent histones [11]. ISH and EdU incorporation showed that these histones are expressed exclusively during S-phase (Fig. 2c). We conclude that the histone cluster described here represents the canonical core histones of *Hydractinia*. The protein and coding sequences of all 19 histones are deposited in GenBank (KX622123-41).

### Common histone variants

**Fig. 4:**
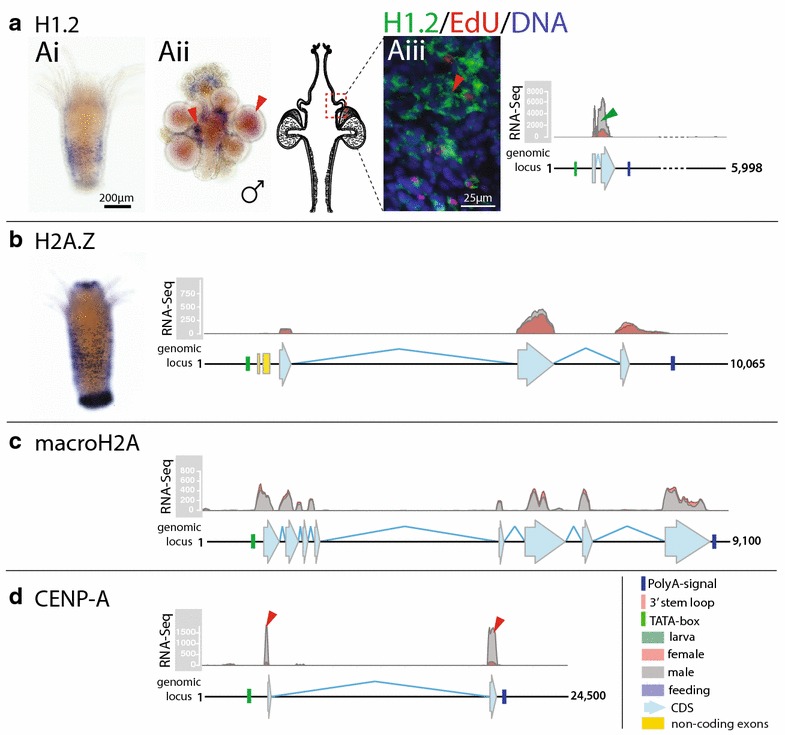
Annotated genomic loci and expression profiles of *Hydractinia echinata* H1.2, H2A.Z, macroH2A and CENP-A. **a** H1.2 expression in feeding (*Ai*) and male sexual polyps (*Aii*). RNA-Seq mapping shows that H1.2 transcripts are found in larva, female, feeding polyps and highly abundant in male polyps (*green wedge*; RNA-Seq track). The annotated genomic locus of H1.2 shows its coding sequence, mapped RNA reads (showing the number of reads mapped), a predicted TATA-boxes and a polyA signal. The gene contains two exons. H1.2 expression is replication-independent, and its transcripts do not exclusively co-localise with EdU-positive S-phase cells (*red wedge*; *Aiii*). **b** H2A.Z expression in feeding polyps. RNA-Seq shows that the gene contains five exons, of which 2 are non-coding. The H2A.Z transcript is abundant in all life stages. The annotated genomic locus of H2A.Z shows its coding sequence, mapped RNA reads (showing the number of reads mapped), a predicted TATA-box and a polyA signal. **c** The annotated genomic locus of macroH2A showing its coding sequence, mapped RNA reads (showing the number of reads mapped), a predicted TATA-box and polyA signal. The gene contains eight exons. **d** The annotated genomic locus of CENP-A shows its coding sequence, mapped RNA reads (showing the number of reads mapped), a predicted TATA-box and a polyA signal. The gene contains two exons

**Fig. 5:**
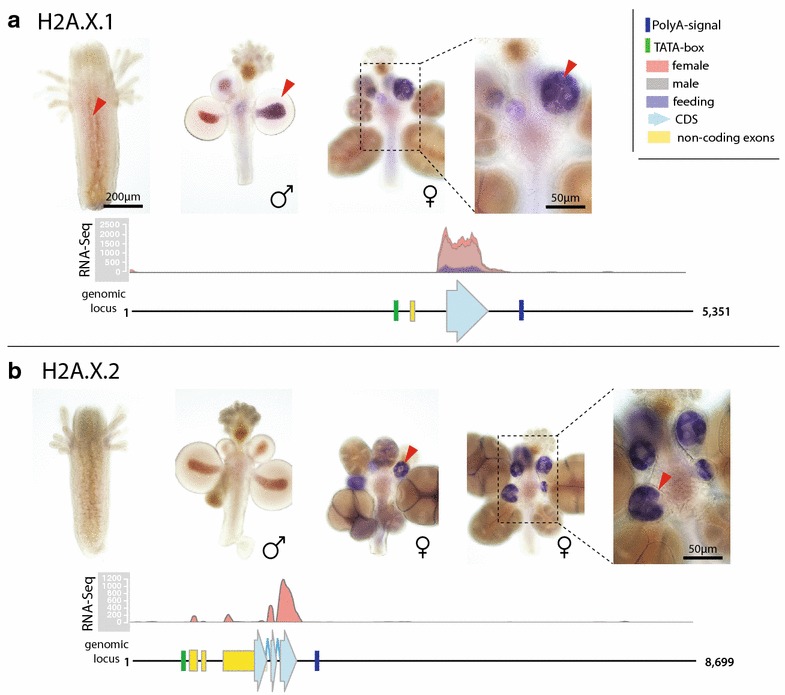
Annotated genomic loci and expression profiles of *Hydractinia echinata* H2A.X.1 and H2A.X.2. **a** H2A.X.1 expression in feeding, male and female sexual polyps (*red wedges*; RNA-Seq mapping track). The annotated genomic locus of H2A.X.1 shows its coding sequence, mapped RNA reads (showing the number of reads mapped), a predicted TATA-boxes and a 3′-UTR stem-loop. The gene contains two exons, and one of them is non-coding. **b** H2A.X.2 expression is restricted to female sexual polyps (*red wedges*; RNA-Seq mapping track) and absent in feeding and male sexual polyps. The annotated genomic locus of H2A.X.2 shows its coding sequence, mapped RNA reads (showing the number of reads mapped), a predicted TATA-box and a polyA signal. The gene contains five exons, two of which are non-coding

**Fig. 6:**
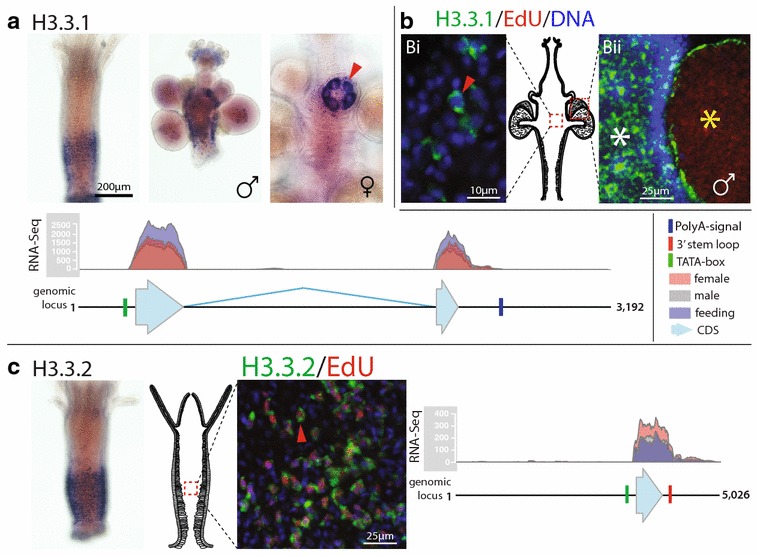
Annotated genomic loci and expression profiles of *Hydractinia echinata* H3.3.1 and H3.3.2. **a** H3.3.1 expression in feeding polyps and male and female sexual polyps. H3.3.1 is highly abundant in female germ cells (red wedge). The annotated genomic locus of H3.3.1 shows its coding sequence, mapped RNA reads (showing the number of reads mapped), a predicted TATA-box and a polyA signal. The gene contains two exons. **b** H3.3.1 expression is replication-independent and its transcripts do not co-localise with EdU-positive S-phase cells in both feeding polyps (*red wedge* in *Bi*) and male sexual polyps (*white asterisk* in *Bii*). Replicating cells do not contain H3.3.1 transcripts (*yellow asterisk* in *Bii*). **c** H3.3.2 expression in feeding polyps. The annotated genomic locus of H3.3.2 shows its coding sequence, mapped RNA reads (showing the number of reads mapped), a predicted TATA-box and a 3′-UTR stem-loop. The gene contains one exon. H3.3.2 expression is replication-dependent, and its transcripts co-localise with EdU-positive S-phase cells (*red wedge*)

The *Hydractinia* genome encodes two additional H1, one CENP-A, two H3.3, two H2A.X, one H2A.Z and one macroH2A histone gene. The genes are found at single genomic loci of various sizes. The variant histone genes contain no introns (H1.3, H3.3.1), one intron (H1.2, H2A.X.1, CENP-A, H3.3.2), four introns (H2A.X.2, H2A.Z) or seven introns (macroH2A) (Figs. 4, 5, 6). RNA-Seq, ISH and EdU incorporation assays indicate that, with the exception of H1.2 and H3.3.1, these genes are replication-independent, consistent with the absence a 3′-UTR stem-loop and the presence of a polyA signal (Figs. 4b–d, 5a, b, 6A). All the common, replication-dependent histones—other than H2A.X.2—can be readily identified in larva, female feeding polyps and male sexual polyps by MS-based proteomics following acid extraction (Additional file 4: S4, Additional file 5: S5).

#### Hydractinia expresses an additional replication-independent linker histone (H1.2) and contains a linker histone pseudogene (H1.3)

We found two additional H1 genes in the *Hydractinia* genome, which we named H1.2 and H1.3. The H1.2 gene contains two exons, lacks a 3′-UTR stem-loop and instead contains a polyA signal indicating replication-independent expression (Fig. 4Ai, Aii). H1.2 is the shortest of the three H1 histones lacking two N-terminal XPKK repeats which are found in the canonical *Hydractinia* H1 counterpart (Additional file 3: S3). RNA-Seq shows that H1.2 is expressed in all colony parts, but transcripts are most abundant in male sexual polyps (green wedge; Fig. 4a). ISH shows that H1.2 is expressed in a band-like pattern in the body column of feeding and sexual polyps and in male gonads. H1.2 is expressed independent of S-phase (Fig. 4Aiii).

The coding sequence of H1.3 is intron-less, contains a 3′-UTR stem-loop and is identical on the nucleotide level to the canonical H1 sequence. However, the 3′-UTR stem-loop includes three mismatches, which are predicted to result in a thermodynamically unstable structure using the ‘RNAfold’ software. A distance matrix generated using k-mer-based alignment-free sequence comparison shows that all *Hydractinia* histone 3′-UTR stem-loops cluster together to the exclusion of the H1.3 stem-loop sequence (Additional file 7: S7). This loss of the stem-loop structure suggests that H1.3 may not be expressed in a replication-dependent manner and we find no 3′-UTR polyA signal or distinct H1.3 reads in RNA-Seq data. We therefore conclude that the H1.3 gene is never expressed and represents a non-functional pseudogene.

#### Hydractinia possesses an oocyte-specific H2A.X variant (H2A.X.2)

We find two H2A.X variants in the genome of *Hydractinia* (see above; Figs. 1 and 5), and H2A.X histones share a canonical SQEY amino acid sequence (consensus SQ[E/D/I][Y/F/L]) at the extreme C terminus [1, 13]. The serine (S) in this consensus sequence is specifically phosphorylated in response to DNA damage [44, 45]. *Hydractinia* H2A.X.1 contains the SQEY C-terminal consensus sequence, while H2A.X.2 ends in SQAY (Additional file 3: S3). H2A.X.1 is expressed in all polyp types and thus likely represents the canonical H2A.X (Fig. 5a), whereas H2A.X.2 is only expressed in female germ cells as shown by RNA-Seq and ISH (Fig. 5b).

#### Hydractinia evolved an additional H3.3 gene (H3.3.2) that is replication-dependent

*Hydractinia* possess two H3.3 variants (Fig. 6). The two H3.3 genes encode identical proteins, but differ on the nucleotide level (77.9 % similar) and their genomic context. The *Hydractinia* H3.3.1 gene is encoded by two exons and possesses a polyA signal (Fig. 6a), suggesting replication-independent expression of this histone, whereas the H3.3.2 gene is encoded by one exon and possesses a 3′-UTR stem-loop (Fig. 6c), implying replication-dependent expression. RNA-Seq shows that both H3.3 variants are expressed in all life stages of *Hydractinia* (Fig. 6a, b). ISH shows their spatial expression in a subset of cells throughout the animal in both feeding and sexual polyps. ISH in conjunction with EdU incorporation confirms that H3.3.1 is expressed independent of replication and is highly expressed in immature gametes of both sexes (Fig. 6a). Conversely, H3.3.2 is expressed in S-phase cells as predicted by its 3′-UTR stem-loop (Fig. 6b).

### Novel histone variants

**Fig. 7:**
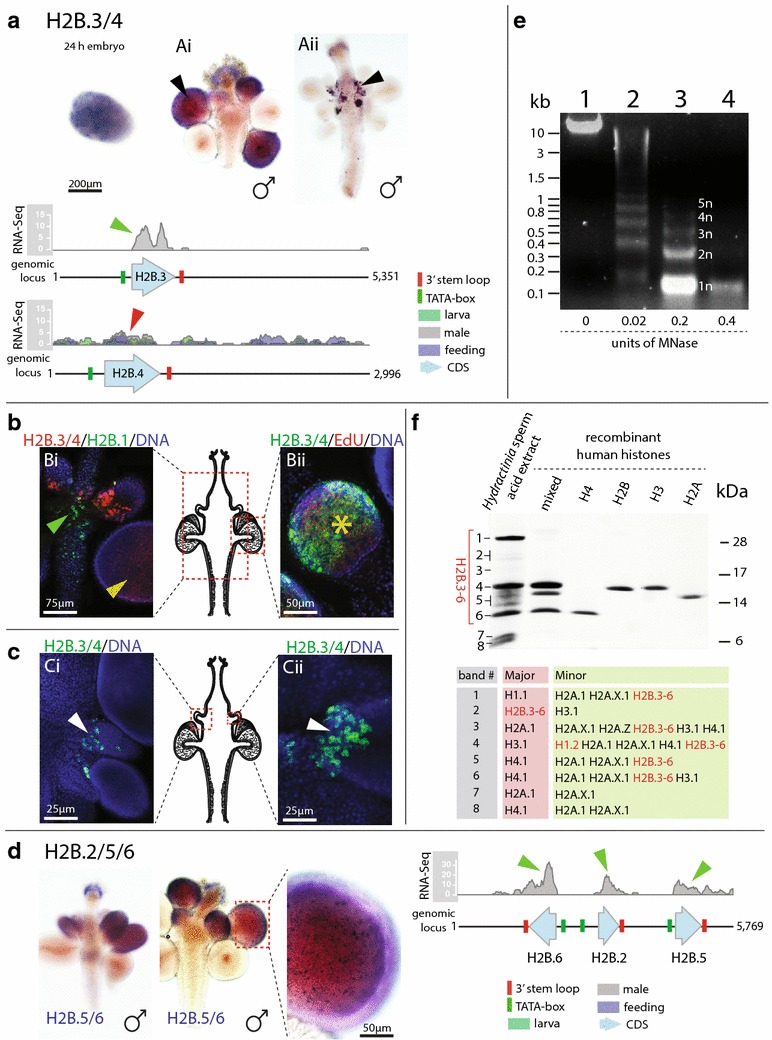
Annotated genomic loci and expression profiles of *Hydractinia echinata* H2B.2-6. **a** H2B.3/4 expression in embryo and male sexual polyps. The annotated genomic loci of H2B.3 and H2B.4 show their coding sequence, mapped RNA reads (showing the number of reads mapped), predicted TATA-boxes and 3′-UTR stem-loops. Both genes contain one exon. RNA-Seq mapping shows that H2B.3 transcripts are only found in male polyps (*green wedge*) and that H2B.4 transcripts are expressed in male sexual polyps, feeding polyps and larva (*red wedge*). Two expression patterns exist, but due to sequence similarities it cannot be determined which pattern is derived from which gene; thus, both expression patterns are shown (*black wedges* in Ai and Aii) using a shared H2B.3/4 annotation. **b** Co-localisations of H2B.1 or S-phase cells with H2B.3/4. Expression patterns of H2B.1 and H2B.3/4 do not overlap (Bi), indicating that H2B.3/4 genes are expressed independent of H2B.1—the *Hydractinia* canonical core H2B. Histone H2B.3/4 expression is replication-dependent, and transcripts co-localise with EdU-positive S-phase cells in male gonads (*yellow asterisk* in *Bii*). **c** H2B.3/4 expression in male polyps using fluorescent probes. The* white wedges* pinpoint an individual cell expressing H2B.3/4 at different magnification (*Ci* and *Cii*). See above for an explanation of the expression patterns in (*Ai)* and (*Aii*). **d** H2B.5/6 expression in male polyps. Endogenous H2B.2 expression could not be determined. Genes for H2B.5 and H2B.6 group with H2B.2 and form a genomic cluster. The annotated genomic locus shows their coding sequence, mapped RNA reads (showing the number of reads mapped), predicted TATA-boxes and 3′-UTR stem-loops. All three genes contain one exon. RNA-Seq mapping shows that their transcripts are only found in male polyps (*green wedges*). **e** Micrococcal nuclease (MNase) digestion of *Hydractinia* sperm cells. *Lane 1* shows sperm genomic DNA extracted in the absence of MNase. *Lanes 2–4* shows sperm genomic DNA extracted after nuclei were subjected to increased concentration of MNase. Nucleosomal DNA bands representing one to five nucleosomal arrays (labelled 1n to 5n) are clearly visible in *lanes 2 and 3*, while in *lane 3* the majority of DNA is present as a mono-nucelosomal (1n) band. No DNA smear or other bands are visible, indicating that the majority of sperm DNA packed by nucleosomes. **f** Coomassie-stained SDS-PAGE of *Hydractinia* sperm acid extracts and recombinant human histones (H2A, H2B, H3 and H4). *Hydractinia* sperm protein bands (labelled with numbers 1–8) were subjected to trypsin digest and consecutive mass spectrometry. Both the major and minor components of each band as determined by mass spectrometry are given. Note, no major band containing H2Bs is apparent; instead, H2B.3-6 proteins are dispersed across the gel (*red bracket*, *red highlight*)

*Hydractinia* has five additional H2B genes (H2B.2-6), of which H2B.3-6 are very similar at the protein levels, making them indistinguishable by protein-based MS (Additional file 5: S5, Additional file 8: S8). At the nucleotide level, H2B.3 is similar to H2B.4, and H2B.5 is similar to H2B.6. Histone H2B.2 is distinct from the other H2B isoforms at both the protein and nucleotide level. As a consequence, the ISH we carried out could only distinguish three subsets of *Hydractinia* H2Bs containing (one) H2B.2, (two) H2B.3/4 or (three) H2B.5/6. RNA-Seq data, however, allowed to precisely distinguish between all *Hydractinia* H2Bs and provided detailed insight in regard to *Hydractinia* H2B expression patterns (Fig. 7a, d).

#### H2B.2/3/5/6 are replication-dependent, sperm-specific histone variants. H2B.4 appears to also be expressed in other tissues

Histone H2B.3/4 mRNA could be detected in embryo and male sexual polyps based on ISH (Fig. 7a). We can show that H2B.3/4 is expressed independent of canonical H2B.1 in gonads (Fig. 7Bi) and confirm that it is expressed in a replication-dependant manner using EdU incorporation (Fig. 7Bii). Histone H2B.3/4 mRNAs were strongly expressed in presumed spermatogonia at the base of immature male gonads (Fig. 7AiI, Bi, C) as well as in developing sperm (Fig. 7Ai, Bii). RNA-Seq data show that only H2B.4 is expressed in larva, feeding and male polyps (Fig. 7a). In summary, based on RNA-Seq, proteomics and ISH data we generated, H2B.2, H2B.3, H2B.5 and H2B.6 specifically expressed in male sexual polyps, while H2B.4 is also expressed in other tissue types. H2B.5 and H2B.6 are expressed exclusively by maturing sperm (Fig. 7d), but it is not clear whether they are co-expressed or sequentially expressed. H2B.2 is exclusive to male sexual polyp in RNA-Seq data (Fig. 7d), but its spatial expression within the polyp remains elusive since its mRNA could not be detected by ISH.

The five variant H2Bs are intron-less and replication-dependent, containing a typical histone 3′-UTR stem-loop and no polyA signals (Figs. 3, 7a, d). Furthermore, H2B.2, H2B.5 and H2B.6 may be functionally linked, as they are organised into a single 5769-bp genomic cluster (Fig. 7d). The histones H2B.3 and H2B.4 are encoded at individual genomic loci (Fig. 7a). H2B.2 is the most divergent H2B variant (Additional file 3: S3, Additional file 5: S5, Additional file 8: S8) with an uncommonly short N terminus lacking the canonical, positively charged amino acids (K or R) (Additional file 6: S6). No specific H2B.2 homologue exists in any other eukaryote. Interestingly, the histone variants H2B.3-6 possess unusual N-terminal extensions that contain a number of conspicuous ‘SPKK’ and ‘SPKR’ repeats. There are five such repeats in H2B.5, six in H2B.3 and H2B6, and seven in H2B.4 (Additional file 8: S8). Such four amino acid repeats have been found before in the N termini of sea urchin H1s and H2Bs, and they are thought to facilitate the dense packaging of sperm DNA in the absence of protamines [14, 16, 42, 43, 46–48]. ‘SPKK’ repeats are also present in *Drosophila* and zebrafish CENP-A [49] as well as angiosperm plant heterochromatin-specific H2A.W [50].

#### Hydractinia lacks sperm nuclear basic proteins (SNBPs) and uses histones to pack its male germinal DNA

Following acid extraction of sperm proteins, eight prominent bands could be observed in SDS-PAGE (Fig. 7f). MS analysis of the tryptic digests of the bands reveals only histones and no protamine-type SNBPs. This by itself is not proof for the absence of protamines and protamine-like proteins as they are insoluble in SDS due to their high arginine content. Therefore, we carried out protein mass spectrometry analysis of total acid extracts from sperm without prior SDS-PAGE. This analysis shows that *Hydractinia* sperm contains only histones and no other major basic proteins (Additional file 9: S9). Furthermore, micrococcal nuclease digestion of *Hydractinia* sperm nuclei clearly demonstrates that sperm chromatin is organised in nucleosomes (Fig. 7e) further corroborating the absence of protamines and SNBPs in sperm. Moreover, database searches against the full transcriptome and draft genome using reciprocal BLAST against NCBI’s NR database also failed to reveal either protamines or protamine-like genes in *Hydractinia*. This is in line with observations made by others that suggest that hydrozoan cnidarians lack protamines entirely [16, 42, 43].

## Discussion

The canonical H1.1, H2A.1, H2B.1, H3.1 and H4.1 genes of *Hydractinia* are present in multiple tandem repeat clusters of approximately 700 copies per haplotype and expressed in S-phase cells, as expected. A similar, partially sequenced cluster containing H3, H4, H2A and H2B was previously described for the coral *Acropora formosa* [51]. Interestingly, the *Hydractinia* canonical histone clusters also contain U1 and U2 snRNAs, a 5S rRNA and an arginine tRNA. While clusters of canonical histones are common in eukaryotes [1], the linkage with other elements is rare. A 5S rRNA was also reported in branchiopod crustaceans and bivalve molluscs [52, 53], and the association of U1 and U2 snRNA genes with 5S rRNA has been observed previously in various eukaryotes [54, 55], but not in the context of a histone gene cluster. The placement of U2 into the canonical histone cluster could be related to its histone-relevant functional role in stem-loop-mediated U7-snRNP-dependent histone 3′-end formation [56]. The placement of U1 in the histone cluster and its role in histone maturation are unclear. Finally, positively charged arginine residues are common in histones, and placing an arginine tRNA gene within the histone cluster may be important in this context.

Similar to other metazoans, *Hydractinia* possesses the common histones variants CENP-A, H2A.X, H2A.Z, macroH2A and H3.3. These are expressed independent of replication and encoded by multi-exon genes outside of the canonical clusters. Expression of these histone variants is consistent with the expected pattern of such ‘replacement’ histones, which are implicated in chromatin repair, remodelling and transcriptional control [6].

Of particular interest are the lineage-specific histone variants. We found that *Hydractinia* possesses additional genes for histone H1 (H1.2 and H1.3), histone H2A.X (H2A.X.2), H2B (H2B.2-6) and H3.3 (H3.3.2). *Hydractinia* H1.2 is expressed throughout all life stages and appears to be upregulated in male polyps, but its role is unknown. H1.3 is not expressed and is likely to be a pseudogene. *Hydractinia* H1.2 upregulation in male polyps suggests that this histone plays a role in sperm development. Distinct H1 variants in males exist in various animals [46]. For example, the sperm-specific H1 variants H1fx and H1B.Sp in *Xenopus* [57], the SpH1 from the sea urchin *Parechinus angulosus* [58] and the mammal H1 variants H1T, HILS1 and H1T2 have all been shown to be involved in spermatogenesis (see references in [46]).

H2A.X.2 is strongly expressed in a replication-independent manner in *Hydractinia* oocytes (Fig. 5a, b). We could not find additional H2A.X genes in other cnidarians outside of the genus *Hydractinia*; thus, the additional H2A.X gene may be the result of a recent gene duplication. The occurrence of specific H2A.X variants in oocytes, eggs and early embryogenesis is rare and has been observed before only in the frog *Xenopus laevis* [15]. Here, the additional H2A.X protein (termed H2A.X-F) is phosphorylated despite the absence of exogenous DNA damage during embryogenesis. During mouse pre-implantation development H2A.X expression was also found to be upregulated [59]. Furthermore, high basal levels of phosphorylated H2A.X were found in mouse embryonic stem cells and associated with global chromatin decondensation rather than DNA damage [60]. Despite these observations, the role of H2A.X in embryogenesis is not yet understood. One hypothesis suggests that H2A.X upregulation is involved in modulation of cellular responses in early cell cycles in rapidly proliferating, externally developing animals [45]. However, the canonical H2A.X of *Hydractinia* (H2A.X.1) does not appear to be upregulated in female polyps when compared to male polyps (Fig. 5A), and thus, it appears that the need for additional H2A.X copies in oocytes of *Hydractinia* is instead provided by a second H2A.X gene (H2A.X.2).

*Hydractinia* H3.3.2 represents another unusual histone, because it is replication-dependent, in contrast to the replication-independent H3.3.1 with identically encoded protein sequence. The occurrence of a replication-dependent H3.3 variant is unusual and has been described previously only in the sea urchin *Strongylocentrotus purpuratus* [47]. Histone H3.3 variants are generally highly conserved and differ from the canonical H3 at four or five specific amino acid positions, notably at position 31, where an alanine (A) is replaced by a serine (S); at positions 87, 89 and 90, where the sequence ‘SAVM’ is replaced by ‘AAIG’; and at position 96 where a cysteine (C) is replaced with a serine (A) [61, 62]. In *Hydractinia*, four out of five of these changes occur, with the last change at position 96 being absent (Additional file 3: S3). Interestingly, some yeast species contain only a single H3 protein sequence, which is highly similar to H3.3 [63]. Based on these data and based on a study that phylogenetically analysed a large number of H3 variants in all eukaryotic supergroups [64], it is evident that H3.3 represents the ancestral protoH3 histone that was most probably present in the last eukaryotic common ancestor (LECA) and that modern, canonical H3 is a derived variant of H3.3. Generally, in cases where canonical H3 and H3.3 co-occur H3.3 is replication-independent and replaces canonical H3 in nucleosomes after nucleosomal displacement during gene transcription to create an epigenetic imprint of transcriptionally active genes [61, 63, 65]. Histone H3.3 is also associated with the repression of telomeric RNA transcription [66] and acts as a maternal factor facilitating the epigenetic reprogramming of the sperm nucleus after fertilisation in mice [67]. In S-phase, H3.3 has been shown to act as a placeholder for CENP-A in centromeres of human cells [68]. The function of the replication-dependent H3.3 in *Hydractinia* and echinoderms is therefore puzzling. Epigenetic H3.3 marks are generally lost during replication and replaced by H3, so the presence of a replication-dependent H3.3 may allow retention of an active transcription mark during S-phase and facilitate gene expression to proceed rapidly and effectively in G2 without the need for *de novo* H3.3 tagging.

Our work corroborates previous studies [16, 42, 43, 46] showing that hydrozoans lack P- and PL-type SNPBs. Instead, four H2B histone variants are expressed either exclusively (H2B.3, H2B.5, H2B.6) or preferentially (H2B.4) in developing sperm. These histones all include SPKK/SPKR motifs at their N termini. We could only find similar H2B histone variants in other hydrozoans, which also appear to lack true protamines, but not in the protamine-like SNBP containing anthozoans (*Nematostella vectensis, Acropora digitifera* and *Exaiptasia pallida* (Aiptasia)) and medusozoas (*Aurelia aurita*, *Chironex fleckeri*) transcriptomes or genomes. Since we find only histones in acid extracts of *Hydractinia* sperm and show that nucleosomes are present in this cell type by MNase assay, we suggest that *Hydractinia* exclusively uses histones to pack its sperm DNA. The absence of protamines in other hydrozoans further suggests that this histone-based type of DNA packaging is a general feature of hydrozoan sperm. It has been shown previously in sea urchin that a similar H2B variant containing these conspicuous SPKK/SPKR motifs is incorporated into nucleosomes but also interacts with linker DNA leading to higher compaction and denser heterochromatin formation [69]. Our data provide additional evidence suggesting that the function of sperm DNA condensation can be performed by N-terminal SPKK/SPKR-containing H2B variants in the absence of protamines.

## Conclusions

This study adds to the limited data available for histone gene complements in metazoans and also provides a framework for studies on the role of histones and their post-translational modifications in cnidarian epigenetics. Our study demonstrates that cnidarians contain rare and unique histone variants. Functional studies on these histones may provide insight into their role in mediating the aforementioned unique cnidarian features.

## Additional files

10.1186/s13072-016-0085-1 **S1.** Histone cluster copy number estimation. (A) 17-mer k-mer counts showing an approx. 20X coverage peak for genomic Illumina paired end read from *Hydractinia echinata* genomic DNA libraries. (B) A second peak and third peak are found at approx. 28,000X and 46,000X coverage. The k-mers from these peaks encode either histone genes or 28S rRNA genes. Based on the 20X coverage of all k-mers this suggests that 1400 copies of the histone cluster and 2300 copies of the 28S rRNA cluster are present in the *Hydractinia echinata* diploid genome (700 histone cluster and 1150 28S rRNA cluster copies per halpoid genome).
10.1186/s13072-016-0085-1 **S2.** List of primers used in this study.
10.1186/s13072-016-0085-1 **S3.** Histone alignments in phylip format used for histone comparison and phylogenetic analysis. Important *Hydractinia echinata* histone variants are highlighted in yellow.
10.1186/s13072-016-0085-1 **S4.** Summary table of all experiments and results.
10.1186/s13072-016-0085-1 **S5.** Graph showing the amino acid sequences of all histones of *Hydractinia echinata* and the corresponding peptides identified by mass-spectrometry following trypsin digest of acid extracted histones from various life stages.
10.1186/s13072-016-0085-1 **S6.** Extension of Fig. 2 showing RNA reads from additional life stages of *Hydractinia echinata* mapped to the H1.1 and core H2A.1, H2B.1, H3.1 and H4.2 genomic cluster.
10.1186/s13072-016-0085-1 **S7.** Tree representing a distance matrix of all *Hydractinia echinata* 3′-UTR stem loop sequences using k-mer based alignment-free sequence comparison. The k-mer based alignment-free sequence comparison was performed using kmacs (http://kmacs.gobics.de/[last accessed: 20/04/2016]).
10.1186/s13072-016-0085-1 **S8.** Annotated alignment showing the SPKK/SPKR repeat number and histone fold domain structure of *Hydractinia echinata* histone H2B.1-6.
10.1186/s13072-016-0085-1 **S9.** Table showing mass-spectrometry results following trypsin digest of acid extracted histones from sperm of *Hydractinia echinata*.
