# Supplementary figures and images for: The cnidarian Hydractinia echinata employs canonical and highly adapted histones to pack its DNA
Source: Epigenetics Chromatin. 2016 Sep 6;9(1):36. doi: 10.1186/s13072-016-0085-1 (PMC5011920; doi:10.1186/s13072-016-0085-1)

**A**

diploid-peak  
at approx. 20X coverage

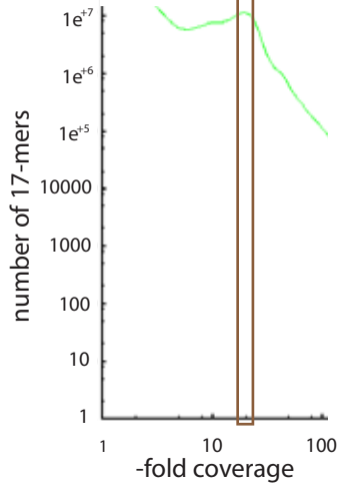**B**

— 17-mer histogram

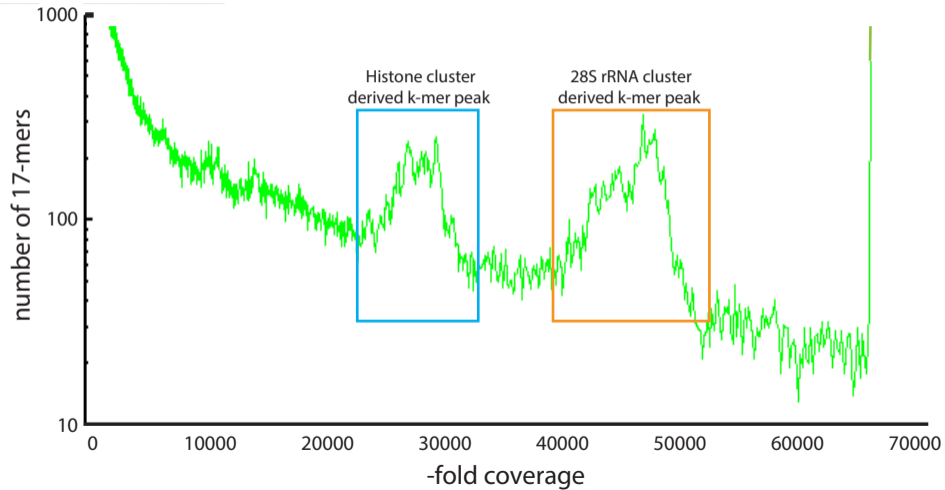

Supplement: Supplementary file 1 — 10.1186/s13072-016-0085-1 S1. Histone cluster copy number estimation. (A) 17-mer k-mer counts showing an approx. 20X coverage peak for genomic Illumina paired end read from Hydractinia echinata genomic DNA libraries. (B) A second peak and third peak are found at approx. 28,000X and 46,000X coverage. The k-mers from these peaks encode either histone genes or 28S rRNA genes. Based on the 20X coverage of all k-mers this suggests that 1400 copies of the histone cluster and 2300 copies of the 28S rRNA cluster are present in the Hydractinia echinata diploid genome (700 histone cluster and 1150 28S rRNA cluster copies per halpoid genome). [file 13072_2016_85_MOESM1_ESM.pdf]

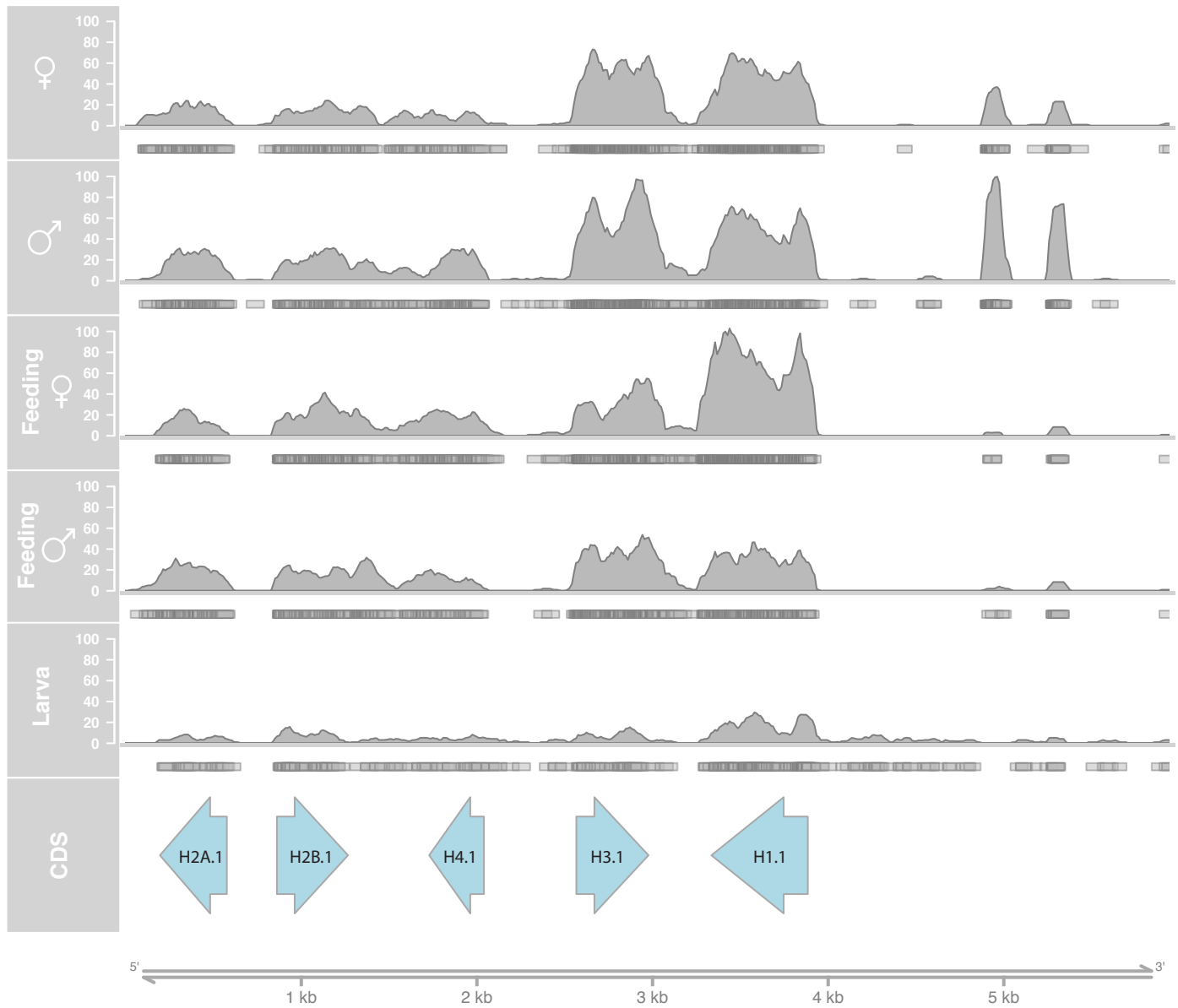

Supplement: Supplementary file 6 — 10.1186/s13072-016-0085-1 S6. Extension of Fig. 2 showing RNA reads from additional life stages of Hydractinia echinata mapped to the H1.1 and core H2A.1, H2B.1, H3.1 and H4.2 genomic cluster. [file 13072_2016_85_MOESM6_ESM.pdf]

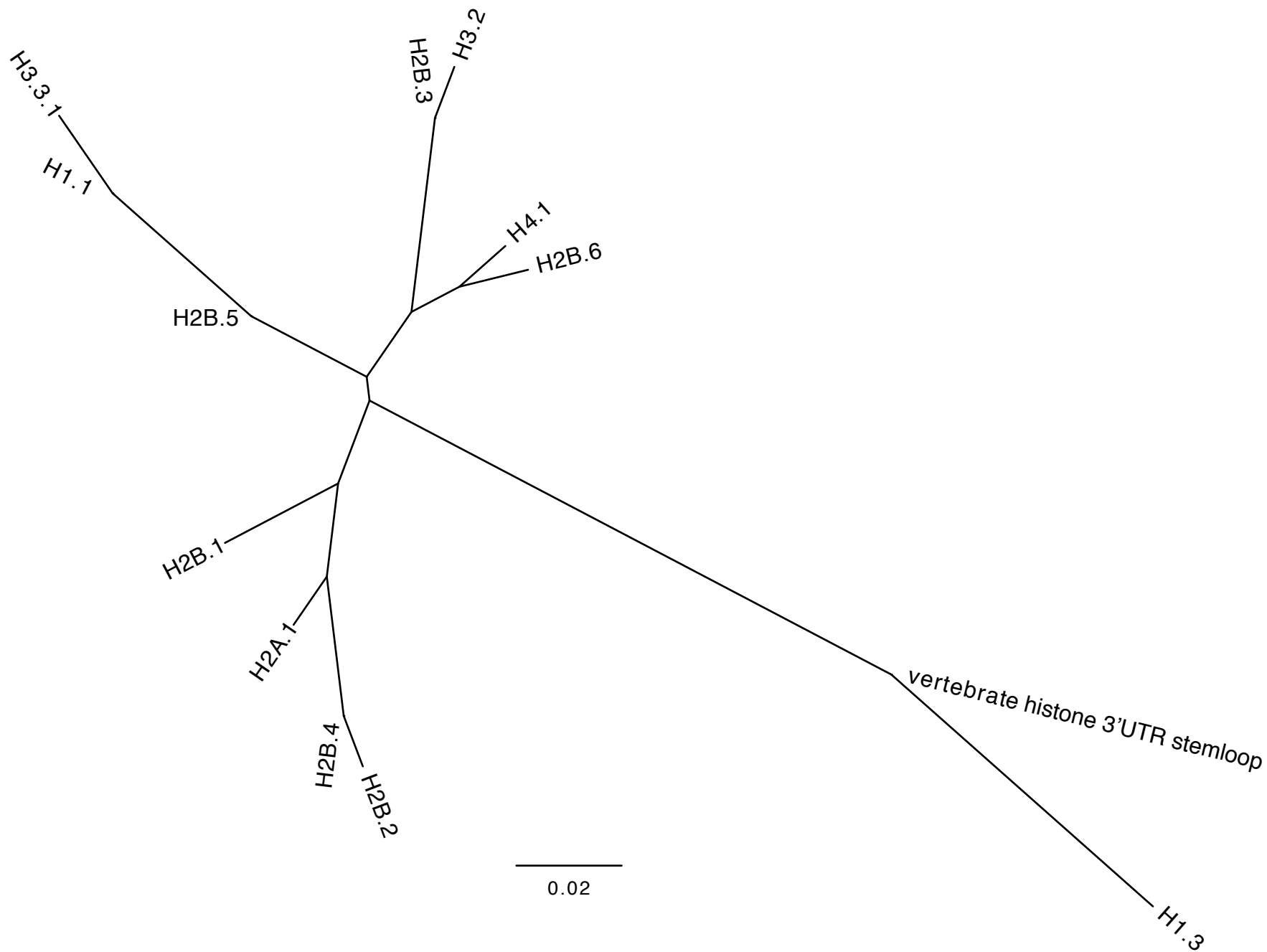

Supplement: Supplementary file 7 — 10.1186/s13072-016-0085-1 S7. Tree representing a distance matrix of all Hydractinia echinata 3′-UTR stem loop sequences using k-mer based alignment-free sequence comparison. The k-mer based alignment-free sequence comparison was performed using kmacs (http://kmacs.gobics.de/[last accessed: 20/04/2016]). [file 13072_2016_85_MOESM7_ESM.pdf]

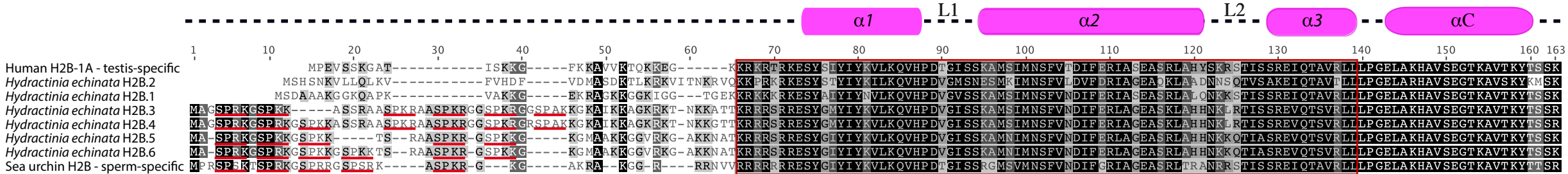

histone-fold

Supplement: Supplementary file 8 — 10.1186/s13072-016-0085-1 S8. Annotated alignment showing the SPKK/SPKR repeat number and histone fold domain structure of Hydractinia echinata histone H2B.1-6. [file 13072_2016_85_MOESM8_ESM.pdf]
