# Supplementary material for: The cnidarian Hydractinia echinata employs canonical and highly adapted histones to pack its DNA
Source: Epigenetics Chromatin. 2016 Sep 6;9(1):36. doi: 10.1186/s13072-016-0085-1 (PMC5011920; doi:10.1186/s13072-016-0085-1)
Supplement: Supplementary file 3 — 10.1186/s13072-016-0085-1 S3. Histone alignments in phylip format used for histone comparison and phylogenetic analysis. Important Hydractinia echinata histone variants are highlighted in yellow. [file 13072_2016_85_MOESM3_ESM.docx]

**H2A,H2B, H3 and H4 alignment (phylip format)**

83 411

NP_003529.1 ------------------------------------------------------------MSGRGKGGKGLGKGGAKRHRKVLRDNIQGITKPAIRRLARRGGVKRIS-------GLIYEETRGVLKVFLENVIRDAVTYTEHAKRKTVTAMDVVYALKRQGRTLYGFGG-------------------------------------------------------------------------------------------------------------------------------------------------------------------------------------------------------------------------------------------------

NP_524352.1 ------------------------------------------------------------MTGRGKGGKGLGKGGAKRHRKVLRDNIQGITKPAIRRLARRGGVKRIS-------GLIYEETRGVLKVFLENVIRDAVTYTEHAKRKTVTAMDVVYALKRQGRTLYGFGG-------------------------------------------------------------------------------------------------------------------------------------------------------------------------------------------------------------------------------------------------

XP_001177078.2 ------------------------------------------------------------MSGRGKGGKGLGKGGAKRHRKVLRDNIQGITKPAIRRLARRGGVKRIS-------GLIYEETRGVLKVFLENVIRDAVTYCEHAKRKTVTAMDVVYALKRQGRTLYGFGG-------------------------------------------------------------------------------------------------------------------------------------------------------------------------------------------------------------------------------------------------

XP_003383775.1 ------------------------------------------------------------MSGRGKGGKGLGKGGAKRHRKILRDNIQGITKPAIRRLARRGGVKRIS-------GLIYEETRGVLKVFLENVIRDAVTYTEHAKRKTVTAMDVVYALKRQGRTLYGFGG-------------------------------------------------------------------------------------------------------------------------------------------------------------------------------------------------------------------------------------------------

XP_004205459.1 ------------------------------------------------------------MSGRGKGGKGLGKGGAKRHRKILRDNIQGITKPAIRRLARRGGVKRIS-------GLIYEETRGVLKVFLENVIRDAVTYTEHAKRKTVTAMDVVYALKRQGRTLYGFGG-------------------------------------------------------------------------------------------------------------------------------------------------------------------------------------------------------------------------------------------------

XP_001618258.1 ------------------------------------------------------------MSGRGKGGKGLGKGGAKRHRKILRDNIQGITKPAIRRLARRGGVKRIS-------GLIYEETRGVLKVFLENVIRDAVTYTEHAKRKTVTAMDVVYALKRQGRTLYGFGG-------------------------------------------------------------------------------------------------------------------------------------------------------------------------------------------------------------------------------------------------

KX622124_Hydractinia_H4.1 ------------------------------------------------------------MSGRGKGGKGLGKGGAKRHRKILRDNIQGITKPAIRRLARRGGVKRIS-------GLIYEETRGVLKVFLENVIRDAVTYTEHAKRKTVTAMDVVYALKRQGRTLYGFGG-------------------------------------------------------------------------------------------------------------------------------------------------------------------------------------------------------------------------------------------------

AIPGENE15694 ------------------------------------------------------------MTGRGKGGKGLGKGGAKRHRKILRDNIQGITKPAIRRLARRGGVKRIS-------GLIYEETRGVLKVFLENVIRDAVTYTEHAKRKTVTAMDVVYALKRQGRTLYGFGG-------------------------------------------------------------------------------------------------------------------------------------------------------------------------------------------------------------------------------------------------

NP_999712.1 ---------------------------MARTKQTARKSTGGKAPRKQLATKAARKSAPATGGVKKPHRYRPGTVALREIRRYQKSTELLIRKLPFQRLVREIAQDFKT--ELRFQSSAVMALQEASEAYLVRLFEDTNLCAIHAKRVTIMPKDIQLARRIRGERA------------------------------------------------------------------------------------------------------------------------------------------------------------------------------------------------------------------------------------------------------

KX622141_Hydractinia_CENP-A -------------MVKTKTTKSPRTLKKAMKSPVVTSTPMTGKGNKKRRRLSSGGRESTDEETPKRRRTHPGTRALKEIRFYQRSTHFLIPKLSFCRLVKETINKSAYRQDFRIQSKALEALQEAAEAFLIRFLEDTNLCAIHARRVTIFPKDMNLVKMLKHEQLFNALEE------------------------------------------------------------------------------------------------------------------------------------------------------------------------------------------------------------------------------------------------

XP_002154677.2 -------------MNKEKKRKSASPLVVANKKRRNTEPSGNNTMNNETTNKTSKSRN----DTTTHRRIKPGIKALKEIRYYQKTDHLLIPKLSFCRVVKEIILSTSK-SDMRIQSQALAALQEATEAYAVRYMQDANMCAIHANRVTIQPKDMKLVAYFDKHL-------------------------------------------------------------------------------------------------------------------------------------------------------------------------------------------------------------------------------------------------------

AIPGENE25970 MVRVSTGNPRKNSRPKQVTTSTPLHRTSVIYHKSPAKKSPAKSPRRTPRKTVTHESSTRRRSGGSHRRYRPGTRALLEIRHYQKTTHLLLRRAPFMRVVRELANKFYQGHELRWQVGALQALQESAEAFLVRLFEDANLCAIHAKRVTIMPKDIQLARRIRGRQDGLG---------------------------------------------------------------------------------------------------------------------------------------------------------------------------------------------------------------------------------------------------

XP_001623949.1 MVRVKSSGGR---------SSSPVRRDPVHYSSKPR------SPSVSGASHGRTPSSPRK-----ARRFRPGTRALMEIKYYQKTTHLLLRKAPFMRVVKEVADRFYTTSELRWQVAALMALQEAAEAFLVRLFEDANLCAIHAKRVTVMPRDIQLARRIRGQDAGLG---------------------------------------------------------------------------------------------------------------------------------------------------------------------------------------------------------------------------------------------------

XP_001177217.2 ---------------------------MARTKQTARKSTGGKAPRKQLATKAARKSAPATGGVKKPHRYRPGTVALREIRRYQKSTELLIRKLPFQRLVREIAQDFKT--ELRFQSSAVMALQEASEAYLVGLFEDTNLCAIHAKRVTIMPKDIQLARRIRGERA------------------------------------------------------------------------------------------------------------------------------------------------------------------------------------------------------------------------------------------------------

XP_003391250.1 ---------------------------MARTKQTARKSTGGKAPRKQLATKAARKSAPATGGVKKPHRYRPGTVALREIRRYQKSTELLIRKLPFQRLVREIAQDFKT--DLRFQSSAVMALQEASEAYLVGLFEDTNLCAIHAKRVTIMPKDIQLARRIRGERA------------------------------------------------------------------------------------------------------------------------------------------------------------------------------------------------------------------------------------------------------

NP_066403.2 ---------------------------MARTKQTARKSTGGKAPRKQLATKAARKSAPATGGVKKPHRYRPGTVALREIRRYQKSTELLIRKLPFQRLVREIAQDFKT--DLRFQSSAVMALQEASEAYLVGLFEDTNLCAIHAKRVTIMPKDIQLARRIRGERA------------------------------------------------------------------------------------------------------------------------------------------------------------------------------------------------------------------------------------------------------

AIPGENE22886 ---------------------------MARTKQTARKSTGGKAPRXQXXTKAARKSAPATGGVKKPHRYRPGTVALREIRRYQKSTELLIRKLPFQRLVREIAQDFKT--DLRFQSSAVMALQEASEAYLVGLFEDTNLCAIHAKRVTIMPKDIQLARRIHAKK-------------------------------------------------------------------------------------------------------------------------------------------------------------------------------------------------------------------------------------------------------

AIPGENE6136 ---------------------------MARTKQTARKSTGGKAPRKQLATKAARXSAPATGGXKKPHRYRPGTVALREIRRYQKSTELLIRKLPFQRLVREIAQDFKT--DLRFQSSAVMALQEASEAYLVGLFEDTNLCAIHAKRVTIMPKDIQLARRIPKKAK------------------------------------------------------------------------------------------------------------------------------------------------------------------------------------------------------------------------------------------------------

AIPGENE3063 ---------------------------MARTKQTARKSTGGKAPRKQLATKAARKSAPATGGVKKPHRYRPGTVALREIRRYQKSTELLIRKLPFQRLVREIAQDFKT--DLRFQSSAVMALQEASEAYLVGLFEDTNLCAIHAKRVTIMPKDIQLARRIHAKK-------------------------------------------------------------------------------------------------------------------------------------------------------------------------------------------------------------------------------------------------------

XP_001618211.1 ---------------------------MARTKQTARKSTGGKAPRKQLATKAARKSAPATGGVKKPHRYRPGTVALREIRRYQKSTELLIRKLPFQRLVREIAQDFKT--DLRFQSSAVMALQEASEAYLVGLFEDTNLCAIHAKRVTIMPKDIQLARRIRGERA------------------------------------------------------------------------------------------------------------------------------------------------------------------------------------------------------------------------------------------------------

NP_724345.1 ---------------------------MARTKQTARKSTGGKAPRKQLATKAARKSAPATGGVKKPHRYRPGTVALREIRRYQKSTELLIRKLPFQRLVREIAQDFKT--DLRFQSSAVMALQEASEAYLVGLFEDTNLCAIHAKRVTIMPKDIQLARRIRGERA------------------------------------------------------------------------------------------------------------------------------------------------------------------------------------------------------------------------------------------------------

KX622127_Hydractinia_H3 ---------------------------MARTKQTARKSTGGKAPRKQLATKAARKSAPATGGVKKPHRYRPGTVALREIRRYQKSTELLIRKLPFQRLVREIAQDFKT--DLRFQSTAVMALQEASEAYLVGLFEDTNLCAIHAKRVTIMPKDIQLARRIRGERA------------------------------------------------------------------------------------------------------------------------------------------------------------------------------------------------------------------------------------------------------

XP_002164221.1 ---------------------------MARTKQTARKSTGGKAPRKQLATKAARKSAPATGGVKKPHRYRPGTVALREIRRYQKSTELLIRKLPFQRLVREIAQDFKT--DLRFQSTAVMALQEASEAYLVGLFEDTNLCAIHAKRVTIMPKDIQLARRIRGERA------------------------------------------------------------------------------------------------------------------------------------------------------------------------------------------------------------------------------------------------------

XP_003384122.1 ---------------------------MARTKQTARKSTGGKAPRKQLATKAARKSAPATGGVKKPHRYRPGTVALREIRRYQKSTELLIRKLPFQRLVREIAQDFKT--DLRFQSSAVAALQEASEAYLVGLFEDTNLCAIHAKRVTIMPKDIQLARRIRGERA------------------------------------------------------------------------------------------------------------------------------------------------------------------------------------------------------------------------------------------------------

NP_001013721.2 ---------------------------MARTKQTARKSTGGKAPRKQLATKAARKSTPSTCGVK-PHRYRPGTVALREIRRYQKSTELLIRKLPFQRLVREIAQDFNT--DLRFQSAAVGALQEASEAYLVGLLEDTNLCAIHAKRVTIMPKDIQLARRIRGERA------------------------------------------------------------------------------------------------------------------------------------------------------------------------------------------------------------------------------------------------------

XP_001623766.1 ---------------------------MARTKQTARKSTGGKAPRKQLATKAARKSAPSTGGVKKPHRYRPGTVALREIRRYQKSTELLIRKLPFQRLVREIAQDFKT--DLRFQSAAIGALQEAAEAYLVGLFEDTNLCAIHAKRVTIMPKDIQLARRIRGERA------------------------------------------------------------------------------------------------------------------------------------------------------------------------------------------------------------------------------------------------------

AIPGENE4053 ---------------------------MLFRKQTARKSTGGKAPRKQLATKAARKSAPSTGGVKKPHRYRPGTVALREIRRYQKSTELLIRKLPFQRLVREIAQDFKT--DLRFQSAAIGALQEAAEAYLVGLFEDTNLCAIHAKRVTIMPKDIQLARRIRGERA------------------------------------------------------------------------------------------------------------------------------------------------------------------------------------------------------------------------------------------------------

XP_002154470.1 ---------------------------MARTKQTARKSTGGKAPRKQLATKAARKSAPSTGGVKKPHRYRPGTVALREIRRYQKSTELLIRKLPFQRLVREIAQDFKT--DLRFQSAAIGALQEAAEAYLVGLFEDTNLCAIHAKRVTIMPKDIQLARRIRGERA------------------------------------------------------------------------------------------------------------------------------------------------------------------------------------------------------------------------------------------------------

KX622125_KX622126_Hydractinia_H3.3.1/2 ---------------------------MARTKQTARKSTGGKAPRKQLATKAARKSAPSTGGVKKPHRYRPGTVALREIRRYQKSTELLIRKLPFQRLVREIAQDFKT--DLRFQSAAIGALQEAAEAYLVGLFEDTNLCAIHAKRVTIMPKDIQLARRIRGERA------------------------------------------------------------------------------------------------------------------------------------------------------------------------------------------------------------------------------------------------------

NP_002098.1 ---------------------------MARTKQTARKSTGGKAPRKQLATKAARKSAPSTGGVKKPHRYRPGTVALREIRRYQKSTELLIRKLPFQRLVREIAQDFKT--DLRFQSAAIGALQEASEAYLVGLFEDTNLCAIHAKRVTIMPKDIQLARRIRGERA------------------------------------------------------------------------------------------------------------------------------------------------------------------------------------------------------------------------------------------------------

NP_511095.1 ---------------------------MARTKQTARKSTGGKAPRKQLATKAARKSAPSTGGVKKPHRYRPGTVALREIRRYQKSTELLIRKLPFQRLVREIAQDFKT--DLRFQSAAIGALQEASEAYLVGLFEDTNLCAIHAKRVTIMPKDIQLARRIRGERA------------------------------------------------------------------------------------------------------------------------------------------------------------------------------------------------------------------------------------------------------

XP_003386893.1 ---------------------------MARTKQTARKSTGGKAPRKQLATKAARKSAPSTGGVKKPHRYRPGTVALREIRRYQKSTELLIRKLPFQRLVREIAQDFKT--DLRFQSAAIGALQEASEAYLVGLFEDTNLCAIHAKRVTIMPKDIQLARRIRGERA------------------------------------------------------------------------------------------------------------------------------------------------------------------------------------------------------------------------------------------------------

XP_791401.1 ---------------------------MARTKQTARKSTGGKAPRKQLATKAARKSAPSTGGVKKPHRYRPGTVALREIRRYQKSTELLIRKLPFQRLVREIAQDFKT--ELRFQSAAIGALQEASEAYLVGLFEDTNLCAIHAKRVTIMPKDIQLARRIRGERA------------------------------------------------------------------------------------------------------------------------------------------------------------------------------------------------------------------------------------------------------

KX622133_Hydractinia_H2B.1 --------------------------MSHSNKVLLQLKVFVHDFVDMASDKTLKRKVITNKRVQKKPRKRK--ESYSTYIYKILKQVHPDVGMSNESMKIMNSFVLDVFDRIAGEAQKLAADNNSQTVSAKEIQTAVTLLLPGELARHAVSEGTKAVSKYKMSK-------------------------------------------------------------------------------------------------------------------------------------------------------------------------------------------------------------------------------------------------------

XP_002162131.1 -----MAGSPKKGSPRKASPKRGGSPKKTSPRKASPHRSSRPASPNKKAHKGKKAIKAIHKKNKKSHHRRKGKQSYGIYIYRVLKQVHPDVGISSKAMSIMNSFVNDIFERIAGESSKLAVHNKKMTISSREVQTSVRLILPGELAKHAVSEGTKAVTKYSSSK-------------------------------------------------------------------------------------------------------------------------------------------------------------------------------------------------------------------------------------------------------

KX622131_Hydractinia_H2B.3 -----MAGSPRKGSPKKASSRAASPKRAASPKRGGSPKRGGSPAKKGKAIKKAGKRKTNKKATTKRRRSRR--ESYGMYIYKVLKQVHPDVGISSKAMSIMNSFVNDIFERLAGEASKLAHHNKLRTISSREVQTSVRLLLPGELAKHAVSEGTKAVTKYTSSR-------------------------------------------------------------------------------------------------------------------------------------------------------------------------------------------------------------------------------------------------------

KX622130_Hydractinia_H2B.4 MAGSPRKGSPRKGSPKKASSRAASPKRAASPKRGGSPKRGRSPAKKGKAIKKAGKRKTNKKGTTKRRRSRR--ESYGMYIYKVLKQVHPDVGISSKAMSIMNSFVNDIFERIAGEASKLAHHNKLRTISSREVQTSVRLLLPGELAKHAVSEGTKAVTKYTSSR-------------------------------------------------------------------------------------------------------------------------------------------------------------------------------------------------------------------------------------------------------

KX622129_Hydractinia_H2B.5 ------MASPRKGSPKKGSPKKTS--RAASPKRG-SPKKG-----KGMAAKKGGVRKGAKKNATKRRRSRR--ESYGIYIYKVLKQVHPDVGISSKAMNIMNSFVNDIFERLAGEASRLAHHNKKQTIASREVQTSVRLLLPGELAKHAVSEGTKAVTKYTSSK-------------------------------------------------------------------------------------------------------------------------------------------------------------------------------------------------------------------------------------------------------

KX622128_Hydractinia_H2B.6 -MASPRKGSPRKGSPKKGSPKKTS--RAASPKRG-SPKKG-----KGMAAKKGGVRKGAKKNATKRRRSRR--ESYGIYIYKVLKQVHPDVGISSKAMNIMNSFVNDIFERLAGEASRLAHHNKKQTIASREVQTSVRLLLPGELAKHAVSEGTKAVTKYTSSK-------------------------------------------------------------------------------------------------------------------------------------------------------------------------------------------------------------------------------------------------------

XP_781938.1 --------------------------MPPKGAAAKGEKKA----------VKSKSMAVGRTGDKKRRRRRL--ESYNIYIYKVLKQVHPDTGISSKAMSIMNSFVNDIFERIASEASRLAQYNKKSTISSREVQTAVRLLLPGELAKHAVSEGTKAVTKYTTSR-------------------------------------------------------------------------------------------------------------------------------------------------------------------------------------------------------------------------------------------------------

XP_003724619.1 --------------------------MPAKQTSGKGAKKA----------GKAKGRPSGAS--KTRRRKRK--ESYGIYIYKVLKQVHPDTGISSKAMSIMNSFVNDVFQRIAGEASRLALYNKKSTISSREIQTAVRLLLPGELAKHAVSEGTKAVTKYTTSK-------------------------------------------------------------------------------------------------------------------------------------------------------------------------------------------------------------------------------------------------------

NP_999721.1 ----------MPRSPSKTSPRKGSPRRGSPSRKASPKRGG--------KGAKRAGKGGRRRNVVRRRRRRR--ESYGIYIYKVLKQVHPDTGISSRGMSVMNSFVNDIFGRIAGEASRLTRANRRSTISSREIQTAVRLLLPGELAKHAVSEGTKAVTKYTTSR-------------------------------------------------------------------------------------------------------------------------------------------------------------------------------------------------------------------------------------------------------

AIPGENE1752 ----------------------------MPPKPAGVGKKG----------EKKAXKKAALGDKKKRSRRRK--ETYSIYIYKVMKQVHPDTGISSKAMSIMNSFVQDVFERIAGEASRLAHYNKKSTITSREIQTAVRLLLPGELAKHAVSEGTKAVTKYTSSK-------------------------------------------------------------------------------------------------------------------------------------------------------------------------------------------------------------------------------------------------------

XP_001617661.1 ----------------------------MPPK--SGKKPE----------ASKGKKNVVAGDKKKRKGRRK--ESYAIYIYKVLKQVHPDTGISSKAMGIMNSFVNDIFERIAAESSRLAHYNKKSTISSREIQTAIRLLLPGELAKHAVSEGTKAVTKYTSSK-------------------------------------------------------------------------------------------------------------------------------------------------------------------------------------------------------------------------------------------------------

XP_001626726.1 ----------------------------MPPKIKAVKKGG----------KKQA------GDKKKRNRRRK--ESYAIYIYKVLRQVHPDTGISSKAMGIMNSFVNDIFERIAGEASRLAHYNKKHTISSREVQTAVRLLLPGELAKHAVSEGTKAVTKYTSSK-------------------------------------------------------------------------------------------------------------------------------------------------------------------------------------------------------------------------------------------------------

XP_001623232.1 ----------------------------MPPKAAVAAAKV----------VKKPGKKQ--GDKKKKNRKRK--ESYAIYIYKVLKQVHPDTGISSKAMGIMNSFVNDIFERIAGEASRLAHYNKRSTISSREVQTAVRLLLPGELAKHAVSEGTKAVTKYTSSK-------------------------------------------------------------------------------------------------------------------------------------------------------------------------------------------------------------------------------------------------------

NP_733759.1 -------------------------MPEVSSKGATISKKG----------FKKAVVKTQKKEGKKRKRTRK--ESYSIYIYKVLKQVHPDTGISSKAMSIMNSFVTDIFERIASEASRLAHYSKRSTISSREIQTAVRLLLPGELAKHAVSEGTKAVTKYTSSK-------------------------------------------------------------------------------------------------------------------------------------------------------------------------------------------------------------------------------------------------------

NP_003510.1 -------------------------MPEL-AKSAPAPKKG----------SKKAVTKAQKKDGKKRKRSRK--ESYSVYVYKVLKQVHPDTGISSKAMGIMNSFVNDIFERIASEASRLAHYNKRSTITSREIQTAVRLLLPGELAKHAVSEGTKAVTKYTSSK-------------------------------------------------------------------------------------------------------------------------------------------------------------------------------------------------------------------------------------------------------

NP_066407.1 -------------------------MPEP-TKSAPAPKKG----------SKKAVTKAQKKDGKKRKRSRK--ESYSVYVYKVLKQVHPDTGISSKAMGIMNSFVNDIFERIAGEASRLAHYNKRSTITSREIQTAVRLLLPGELAKHAVSEGTKAVTKYTSSK-------------------------------------------------------------------------------------------------------------------------------------------------------------------------------------------------------------------------------------------------------

NP_003518.2 -------------------------MPDP-AKSAPAPKKG----------SKKAVTKAQKKDGKKRKRSRK--ESYSIYVYKVLKQVHPDTGISSKAMGIMNSFVNDIFERIAGEASRLAHYNKRSTITSREIQTAVRLLLPGELAKHAVSEGTKAVTKYTSSK-------------------------------------------------------------------------------------------------------------------------------------------------------------------------------------------------------------------------------------------------------

NP_001019770.1 -------------------------MPDP-AKSAPAPKKG----------SKKAVTKVQKKDGKKRKRSRK--ESYSVYVYKVLKQVHPDTGISSKAMGIMNSFVNDIFERIAGEASRLAHYNKRSTITSREIQTAVRLLLPGELAKHAVSEGTKAVTKYTSSK-------------------------------------------------------------------------------------------------------------------------------------------------------------------------------------------------------------------------------------------------------

NP_724342.1 ----------------------------MPPKTSGKAAKK----------AGKAQKNITKT-DKKKKRKRK--ESYAIYIYKVLKQVHPDTGISSKAMSIMNSFVNDIFERIAAEASRLAHYNKRSTITSREIQTAVRLLLPGELAKHAVSEGTKAVTKYTSSK-------------------------------------------------------------------------------------------------------------------------------------------------------------------------------------------------------------------------------------------------------

XP_003391252.1 ----------------------------MPPKVSAKGAKK----------AGKAKAARSG--DKKKKRRRK--ESYSIYIYKVLKQVHPDTGVSSKAMSIMNSFVNDIFERIAAEASRLAHYNKRSTITSREIQTAVRLLLPGELAKHAVSEGTKAVTKYTSSK-------------------------------------------------------------------------------------------------------------------------------------------------------------------------------------------------------------------------------------------------------

XP_003382971.1 ---------------------------MPGKVASKKGEKK----------AATKTPKPTGDKTKKRRRVRK--ESYSIYIYKVLKQVHPETGISSKAMSIMNSFVNDIFEKIATEASRLAQYNKKSTITSREIQTSVRLWLPGELSKHAVSEGTKAVTKYTSSK-------------------------------------------------------------------------------------------------------------------------------------------------------------------------------------------------------------------------------------------------------

KX622133_Hydractinia_H2B ------------------MSDAAAKGGKQAPKVAKKGEKR----------AGKKGGKIGGTGEKKRKKKRK--ESYAIYIYNVLKQVHPDVGVSSKAMSIMNSFVNDIFERIASEASRLALQNKKSTISSREIQTAVRLLLPGELAKHAVSEGTKAVTKYTSSK-------------------------------------------------------------------------------------------------------------------------------------------------------------------------------------------------------------------------------------------------------

XP_002158733.2 ------------------MSDAPKTGGKQAPKVAKKGEKR----------AGKKGGKIAGTGDKKRKKKRR--ESYAIYIYNVLKQVHPDVGVSSKAMSIMNSFVNDIFERIASEASRLALQNKKSTISSREIQTAVRLLLPGELAKHAVSEGTKAVTKYTSSK-------------------------------------------------------------------------------------------------------------------------------------------------------------------------------------------------------------------------------------------------------

KX622134_Hydractinia_H2A.Z -----------------------------MAGGKAGKDS-KPKTKSTSRSARAGLQFPVGRIHRYLKSRSTNKGRVGATAAVYSAAILEYLTAEVLELAGNASKDLKVKRISPRHLQLAIRGDEELDLLIK-ATIAGGGVIPHIHKSLIGKKGAKPT--------------------------------------------------------------------------------------------------------------------------------------------------------------------------------------------------------------------------------------------------------------

XP_004205771.1 -----------------------------MAGGKAGKDS-KPKTKSTSRSARAGLQFPVGRIHRYLKSRSTNKGRVGATAAVYSAAILEYLTAEVLELAGNASKDLKVKRISPRHLQLAIRGDEELDLLIK-ATIAGGGVIPHIHKSLMNKKTAKQN--------------------------------------------------------------------------------------------------------------------------------------------------------------------------------------------------------------------------------------------------------------

NP_002097.1 -----------------------------MAGGKAGKDSGKAKTKAVSRSQRAGLQFPVGRIHRHLKSRTTSHGRVGATAAVYSAAILEYLTAEVLELAGNASKDLKVKRITPRHLQLAIRGDEELDSLIK-ATIAGGGVIPHIHKSLIGKKGQQKTV-------------------------------------------------------------------------------------------------------------------------------------------------------------------------------------------------------------------------------------------------------------

NP_524519.1 -----------------------------MAGGKAGKDSGKAKAKAVSRSARAGLQFPVGRIHRHLKSRTTSHGRVGATAAVYSAAILEYLTAEVLELAGNASKDLKVKRITPRHLQLAIRGDEELDSLIK-ATIAGGGVIPHIHKSLIGKKEETVQDPQRKGNVILSQAY------------------------------------------------------------------------------------------------------------------------------------------------------------------------------------------------------------------------------------------------

XP_001638352.1 -----------------------------MAGGKAGKDS-KAKAKAVSRSARAGLQFPVGRIHRHLKNRTTSHGRVGATAAVYSAAILEYLTAEVLELAGNASKDLKVKRITPRHLQLAIRGDEELDSLIK-ATIAGGGVIPHIHKSLIGKKGANKPT-------------------------------------------------------------------------------------------------------------------------------------------------------------------------------------------------------------------------------------------------------------

NP_001116980.1 -----------------------------MAGGKAGKDSGKAKAKAVSRSARAGLQFPVGRIHRHLKNRTTSHGRVGATAAVYSAAILEYLTAEVLELAGNASKDLKVKRITPRHLQLAIRGDEELDSLIK-ATIAGGGVIPHIHKSLIGKKGSQKAT-------------------------------------------------------------------------------------------------------------------------------------------------------------------------------------------------------------------------------------------------------------

AIPGENE11701 -----------------------------MAGGKAGKDS-KAKAKAVSRSARAGLQFPVGRIHRHLKNRTTSHGRVGATAAVYSAAILEYLTAEVLELAGNASKDLKVKRITPRHLQLAIRGDEELDSLIK-ATIAGGGAF------------------------------------------------------------------------------------------------------------------------------------------------------------------------------------------------------------------------------------------------------------------------------

XP_781668.1 ----------------------MSAKGGARGVHSGRGGKTRGKS--TSRSAKAGVLFPVGRMDRYLR-MSTHHYRIGSGAPVYLAAVIEYLTAEILELAGNAARDNKKARVTPRHILLAVANDEELHHLLKNVTIASGGVLPQIHPELLMKKRGSKAKSVFDFGQKTPAAVPVPKKPKTPAEKKQ-LPAVKKPVVKKAAL--TPKPNAAVSKGKTISGKSILGEKKLFLGQKLTVVKADLTEITADALVHPTNSTYAMAGEVGSALEKVGGRAFVEEVAKLRAAQS-LDISGAAICPAHNLPAKYVIHVNSPSW---GGANAVSNLEKCIKNCLALADEKNITSIAIPSVSSGRAGFPKQIAAETILRTISHYFVSVMASSLKQIYFVLFDQESVEVYVTELNRLEPDQ--

XP_005272189.1 --------------------------------MSSRGGKKKSTK--TSRSAKAGVIFPVGRMLRYIK-KGHPKYRIGVGAPVYMAAVLEYLTAEILELAGNAARDNKKGRVTPRHILLAVANDEELNQLLKGVTIASGGVLPNIHPELLAKKRGSKGKLEAIITPPPAKKAKSPSQKKPVSKKAGGKKGARKSKKQGEVS--KAASADSTTEGTPADGFTVLSTKSLFLGQKLQVVQADIASIDSDAVVHPTNTDFYIGGEVGNTLEKKGGKEFVEAVLELRKKNGPLEVAGAAVSAGHGLPAKFVIHCNSPVW---GADKCEELLEKTVKNCLALADDKKLKSIAFPSIGSGRNGFPKQTAAQLILKAISSYFVSTMSSSIKTVYFVLFDSESIGIYVQEMAKLDAN---

KX622123_Hydractinia_macroH2A -------------------------------MSGRG----KSKAQRVSISTRAGTIFPVSRIRRYLK-GCTHHQRIAVGAPIYQAAVMEYLSAEILELAGNAARDNKRTRITPRHILLAVANDEELNKLLKNVTIPAGGVMPHIQPELLKRKDGGKFVVPKNDAAVRAALQKAKNAGIQKAKNKPKPVVKAKAPVASKSPVKKVTTPKKKAESKGSDSIAVLSEKTLFLGQKLTIVQGNMESLKCDALVHPTNATFNTTGGVGAALLKVGGEDLKKNIIALHESHGDLAYATALIGEAPNLQAKHIIHVYSPVW--GKG-KAEDDLETVVKNALTLADEKNLATIAFPSIGSGVNQFPKQTAAQTILKAISNYFVTVVTSSLRQIYFVLHDMESIGVYSLELARLETSENK

XP_002154422.2 -------------------------------MSGRG----KNKTQRMTMSSRAGTLFPVARIRRYLR-GCTSKMRVAVGAPVYQAAVMEYLSAEILELAGNAARDNKRNRITPRHILLAIANDEELNKLLKNVTIPSGGVMPHIQPELLKKKDGGKFVVS-NQEKFKPQVLKPLSPGIPKKKKIEKEFVKTATKKALEASTSKKVEKPKKTNVKSDPVFAVLSEKNLYLGQKLTVVQGNIAEMKCDALIHPTNATFNTTGEVGSALLKVGGEELKKAISALHSSHGDLAMSSALLGDSVNLKAKHIIHVHSPTY--VSGGSSEEDLENVVKNALTLADEKNVAVIAFPSIGSGINSFPKQLAAQTILKAISNYFVTVVSSSLKQIYFVLYDMESIGVYTTELAKLG-----

AIPGENE4954 -------------------------------MSARGGKAAKRGKDRMSRSAKAGLQFPVSRVHRYLR-QVTHHYRIASGAPVYQAAVMEYLTAEILELAGNAARDNKKSRIIPRHILLAVANDEELHKLLKGVTIASGGVLPNIHPELLKKRRGGKLVAPEDLPPKKPKQTKEAPXXSPSKG-------KKAPAKPAPPSKGKGKPRGKVASKGPGEGISVLTEKVLFLGQHLTVVQGNIENIEADAIVHPTDGKFTLKSEVGKVLSKIGGDDLKKEIKKLSDSKSDFAVEEAATXPAANMKVSKILHVHCPTYNSAEEEQSTESLMKALKNALAVADDENLKTLAIPSIGTGTFKFPKEQAAQAALKAISNYFVSAMASSIRQVYFALDDMESIGIYTIELARLD-----

XP_001637578.1 -------------------------------MSARGGKAAKRAK-AVSRSAKAGLQFPVSRVHRYLR-KCTHHYRISAAAPVYQAAVMEYLTAEILELAGNAARDNKKTRIIPRHILLAVANDEELHKLLKGVTIASGGVLPNIHPELLKKRKGGKLVSPEELKSKKPKPAPPPS-----------------PKKPVSSKKGRGK-----ADKGPGDGFSVLSEKTLFLGQKLTVVQGDIAAIDADAVVLPTNAKFKLEGEVGEALKKAGGKEFKDEIKKLSEDNGDLALLDAAICDGHNFPAAYVISLHSPVY-SEDSTTASDDLEKAVKNVLTIADEKNLKILAIPSIGTGSNKYPKDLAAQVTLKAISNYFVSAMASSLKQIYFVLSDPENIGMYTMELARLDS----

XP_001623230.1 -----------------------------MSGRGKGG-KSRAKG--KSRSARAGLQFPVGRVHRFLR-KGNYAERVGAGAPVYMAAVLEYLSAEILELAGNAARDNKKTRIIPRHLQLAVRNDEELNKLLGGVTIAQGGVLPNIQAVLLPKRSEKKSK-------------------------------------------------------------------------------------------------------------------------------------------------------------------------------------------------------------------------------------------------------------

NP_999718.1 -----------------------------MSGRGKGAGKARAKA--KSRSARAGLQFPVGRVHRFLR-KGNYAQRVGAGAPVYLAAVLEYLAAEILELAGNAARDNKKTRIIPRHLQLAVRNDEELNKLLSGVTIAQGGVLPNIQAVLLPKKTSKASK-------------------------------------------------------------------------------------------------------------------------------------------------------------------------------------------------------------------------------------------------------------

H2A1A_HUMAN -----------------------------MSGRGKQGGKARAKS--KSRSSRAGLQFPVGRIHRLLR-KGNYAERIGAGAPVYLAAVLEYLTAEILELAGNASRDNKKTRIIPRHLQLAIRNDEELNKLLGGVTIAQGGVLPNIQAVLLPKKTESHHHKAQSK--------------------------------------------------------------------------------------------------------------------------------------------------------------------------------------------------------------------------------------------------------

NP_002096.1 -----------------------------MSGRGKTGGKARAKA--KSRSSRAGLQFPVGRVHRLLR-KGHYAERVGAGAPVYLAAVLEYLTAEILELAGNAARDNKKTRIIPRHLQLAIRNDEELNKLLGGVTIAQGGVLPNIQAVLLPKKTSATVGPKAPSGGKKATQASQEY--------------------------------------------------------------------------------------------------------------------------------------------------------------------------------------------------------------------------------------------

XP_003391253.1 -----------------------------MSGRGK-GGKVKGKS--KTRSSRAGLQFPVGRIHRLLR-KGNYAERVGAGAPVYLAAVLEYLAAEVLELAGNAARDNKKSRIIPRHLQLAIRNDEELNKLLSGVTIAQGGVLPNIQAVLLPKKSEKAASK------------------------------------------------------------------------------------------------------------------------------------------------------------------------------------------------------------------------------------------------------------

NP_724343.1 -----------------------------MSGRGK-GGKVKGKA--KSRSNRAGLQFPVGRIHRLLR-KGNYAERVGAGAPVYLAAVMEYLAAEVLELAGNAARDNKKTRIIPRHLQLAIRNDEELNKLLSGVTIAQGGVLPNIQAVLLPKKTEKKA--------------------------------------------------------------------------------------------------------------------------------------------------------------------------------------------------------------------------------------------------------------

NP_001027366.1 -----------------------------MSGRGK-GGKVKGKA--KSRSDRAGLQFPVGRIHRLLR-KGNYAERVGAGAPVYLAAVMEYLAAEVLELAGNAARDNKKTRIIPRHLQLAIRNDEELNKLLSGVTIAQGGVLPNIQAVLLPKKTEKKA--------------------------------------------------------------------------------------------------------------------------------------------------------------------------------------------------------------------------------------------------------------

AIPGENE4770 -----------------------------MSGRGK-GKPKGTKA--KSRSSKAGLQFPVGRIHXFLR-KGNYAQRVGAGAPVYMAAVLEYLSAEILELAGNAARDNKKTRIVPRHIQLAVRNDEELNKLLSGVTISQGGVLPNIQAVLLPKKTGQGKSKK----MD--SVQSQEF--------------------------------------------------------------------------------------------------------------------------------------------------------------------------------------------------------------------------------------------

XP_001641704.1 -----------------------------MSGRGK-GKINKSKV--KTRSSRAGLQFPVGRIHRFLR-KGNYAERVGAGAPVYMAAVLEYLTAEILELAGNAARDNKKSRIVPRHLQLAVRNDEELNKLLQGVTIAQGGVLPNIQAVLLPKKSNTGGSGKSKKGLG--SSQSQEY--------------------------------------------------------------------------------------------------------------------------------------------------------------------------------------------------------------------------------------------

KX622136_Hydractinia_H2A.X.1 -----------------------------MSGRGK-GGKSKAKA--KSRSSRAGLQFPVGRIHRFLR-RGHYANRVGSGAPVYLAAVLEYLSAEILELAGNAARDNKKARIIPRHLQLAVRNDEELNKLLSGVTIAAGGVLPNIQAVLLPKKTTKGK-----------SSQSQEY--------------------------------------------------------------------------------------------------------------------------------------------------------------------------------------------------------------------------------------------

KX622137_Hydractinia_H2A -----------------------------MSGRGK-GGKAKAKA--KTRSSRAGLQFPVGRVHRFLR-RGHYANRIGSGAPVYLAAVLEYLSAEILELAGNAARDNKKARIIPRHLQLAVRNDEELNKLLSGVTIAAGGVLPNIQAVLLPKKNDKGQKK------------------------------------------------------------------------------------------------------------------------------------------------------------------------------------------------------------------------------------------------------------

XP_004211232.1 -----------------------------MSGRGK-IGKAKAKA--KTRSFRAGLQFPVGRVHRFLR-KGHYANRIGSGAPVYLAAVLEYLSAEILELAGNAARDNKKARIVPRHLQLAVRNDEELNKLLSGVTIASGGVLPNIQAVLLPKKNEKLPKPAAAK--------------------------------------------------------------------------------------------------------------------------------------------------------------------------------------------------------------------------------------------------------

XP_002158325.1 -----------------------------MSGRGK-GGKKSGKS--KTRSSRAGLQFPVGRIHRFLR-KGHYAERIGSGAPVYLAAVLEYLSAEILELAGNAARDNKKSRIVPRHLQLAVRNDEELNKLLSGVTIASGGVLPNIQAVLLPKKTKEPQSK---------SSQSQEY--------------------------------------------------------------------------------------------------------------------------------------------------------------------------------------------------------------------------------------------

KX622135_Hydractinia_H2A.X.2 -----------------------------MSGKGKGKGHLIHHKNRRTRSQMAGVQFPVGRLHRMLK-KGHYADRIGSGAPVYLAAVLEYLTAEILELAGNAARDNKRIRIVPRHLSLAIRNDEELNDLLKGVTIAEGGVLPNIQSALLPKKSMKSSSK------D--GVQSQAY--------------------------------------------------------------------------------------------------------------------------------------------------------------------------------------------------------------------------------------------

**H1 alignment (phylip format)**

10 259

H11_HUMAN ------MSETVPPAPAASAAPEKPLAGKKAKKPAKAAAASKKKPAGPSVSELIVQAASSSKERGGVSLAALKKALAAAGYDVE----KNNSRIKLGIKSLVSKGTLVQTKGTGASGSFKLNKKASS----------------------------VETKPGASKVATKTKATGASKKLKKATG-ASKKSVKTPKKAKKPAATRKSSKNPKKPKTVKPKKVAKSPAKAKAVKPKAAKARVTKPKT-AKPKKAAPKKK----

H11_MOUSE ------MSETAPVAQAASTATEKPAAAKKTKKPAKAAAP-RKKPAGPSVSELIVQAVSSSKERSGVSLAALKKSLAAAGYDVE----KNNSRIKLGLKSLVNKGTLVQTKGTGAAGSFKLNKKA-------------------------------ESKAITTKVSVKAKASGAAKKPKKTAGAAAKKTVKTPKKPKKPAVSKKTSKSPKKPKVVKAKKVAKSPAKAKAVKPKASKAKVTKPKTPAKPKKAAPKKK----

H1 MSDSAVATSASPVAAPPATVEKKVVQKKASGSAGTKAKKASATPSHPPTQQMVDASIKNLKERGGSSLLAIKKYITAT-YKCD--AQKLAPFIKKYLKSAVVNGKLIQTKGKGASGSFKLSASAKKEKDPKAKSKVLSAEKKVQSKKVASKKIGVSSKKTAVGAADKKPKAKKAVATKKTAENKKTEKAKAKDAKKTGIIKSKPAATKAKVTAAKPKAVVAKASKAKPAVSAKPKKTVKKASVSATAKKPKAKTTAAKK

KX622139_Hydractinia_H1.1/3 ----------------------MSEAASPKKVAPKKKPAAKKTADHPKYVDMIKAAIATLKERGGSSRQAITKYIHAN-YKVA---ENSDHHLKMALKRGVTSGDLIQTKGTGASGSFKLGQ-------------------------------------------VKKEKPKKKVAAKKPTAKKPAAKKSTPKKKAAKKSTPKKAAKKPAAKKASAKKPAAKKPTKKPVAKKPAAKKVKKTPKKAKKTAKK--------

NP_999722.1 -------------MADTDAAPAAPAPSTPKKAAKKKASKPKTPASHPKYSDMIASALESLKEKKGSSRQAILKYVKAN-FTVG---DNANVHIKQALKRGVTSGQLRHVKGSGASGSFLLAE-------------------------------------------KTKTPKKAAAKKATPKKKPAAKKTKKPAAKKATKKPAKKPAAKKKVAKPAAKKAAKPVAKKATPKKKVVKKAAKGKGKKK--------------

XP_003391254.1 -------------MTDAAVAPVHKSP------KKKTSAKPKVPAAHPKYVDMIKAAVASLKERGGSSRQAILKYVMAN-YKVGTDLKAVNSRIKNALKNGVKAGTLKQSKGTGAAGSFKLGE-------------------------------------------SKSE--KPSKVKKVAAKKPAAKKASTPKKPKAKKTTTKKTATPKKTKVAGAKKTAAKPKKAAPKKVKTPKKVKTVKPKKASPKKKTAAKKA---

KX622138_Hydractinia_H1.2 ------------------------------------MVAAKKNADHPLFIEMISAAITALKERKGSSRQAIVKYIKAN-YKVG---DNVETVVKMTLKRNIGG-RLVQTKGTGASGSFKLSAP-----------------------------------------AAKKPAAKKPAAKKPAAKKAVAKKPSAKKTPKKTTTATKKKTPKKAAKKTPTKKSPAKKAVKKSKAKKTPTKKSKK-------------------

XP_002159008.2 ----------------------MSEAASPKKIAKKS--APKKPADHAPYKAMIVDAINSLKERKGSSRQAIAKHVKAN-NKVG---DNVDSQVKINLKRMVVAGELVQVKGVGASGSFRVAA-------------------------------------------KPKAAKKKSPVATVKKVKKE--------------------------------------------------------------------------

H1.1_Aiptasia ---------------------------------------------------------------------------------------------------MVAAGKLTQVKGTGASGSFKINKAAVE--------------------------------------KPKK-AKKPAAAKKPAAKKPAAKKPSAKKPAKKPAAKKAKKASPKKAKKPAAKKFTPKKAAAKPAKKPSAKKPTKKPAAKKSPKKAAKKTAKK--

H1.2_Aiptasia ---------------------------------------------------MIKAAILSLKERSGSSRQAIVKYIKAN-YKVG---DNCDVHVKMALKRMVVAGLLNQPKGTGASGSFKVNKEKAE--------------------------------------EKKKPAAKKPAAKKPAAKKPSAKKSAPKKEAKKPAKKAAAKKSTX-KKSKPKKPAAKKPAPKKPAKKPAAKKAAKKPAAKKSGKKGAKK------
