# Supplementary material for: The cnidarian Hydractinia echinata employs canonical and highly adapted histones to pack its DNA
Source: Epigenetics Chromatin. 2016 Sep 6;9(1):36. doi: 10.1186/s13072-016-0085-1 (PMC5011920; doi:10.1186/s13072-016-0085-1)
Supplement: Supplementary file 5 — 10.1186/s13072-016-0085-1 S5. Graph showing the amino acid sequences of all histones of Hydractinia echinata and the corresponding peptides identified by mass-spectrometry following trypsin digest of acid extracted histones from various life stages. [file 13072_2016_85_MOESM5_ESM.pdf]

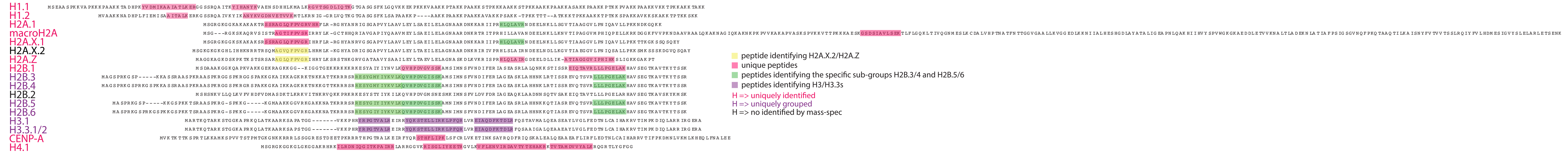
H1.1
H1.2
H2A.1
macroH2A
H2A.X.1
H2A.X.2
H2A.Z
H2B.1
H2B.3
H2B.4
H2B.2
H2B.5
H2B.6
H3.1
H3.3.1/2
CENP-A
H4.1
peptide identifying H2A.X.2/H2A.Z
unique peptides
peptides identifying the specific sub-groups H2B.3/4 and H2B.5/6
peptides identifying H3/H3.3s
H => uniquely identified
H => uniquely grouped
H => no identified by mass-spec
